# Supplementary material for: Trends in the socioeconomic patterning of overweight/obesity in India: a repeated cross-sectional study using nationally representative data
Source: BMJ Open. 2018 Oct 21;8(10):e023935. doi: 10.1136/bmjopen-2018-023935 (PMC6196932; doi:10.1136/bmjopen-2018-023935)

<u>Appendix</u>

Figure A1. Predicted prevalence* of overweight/obesity in India by Education (using South Asian BMI cut-offs) (1998-2016)

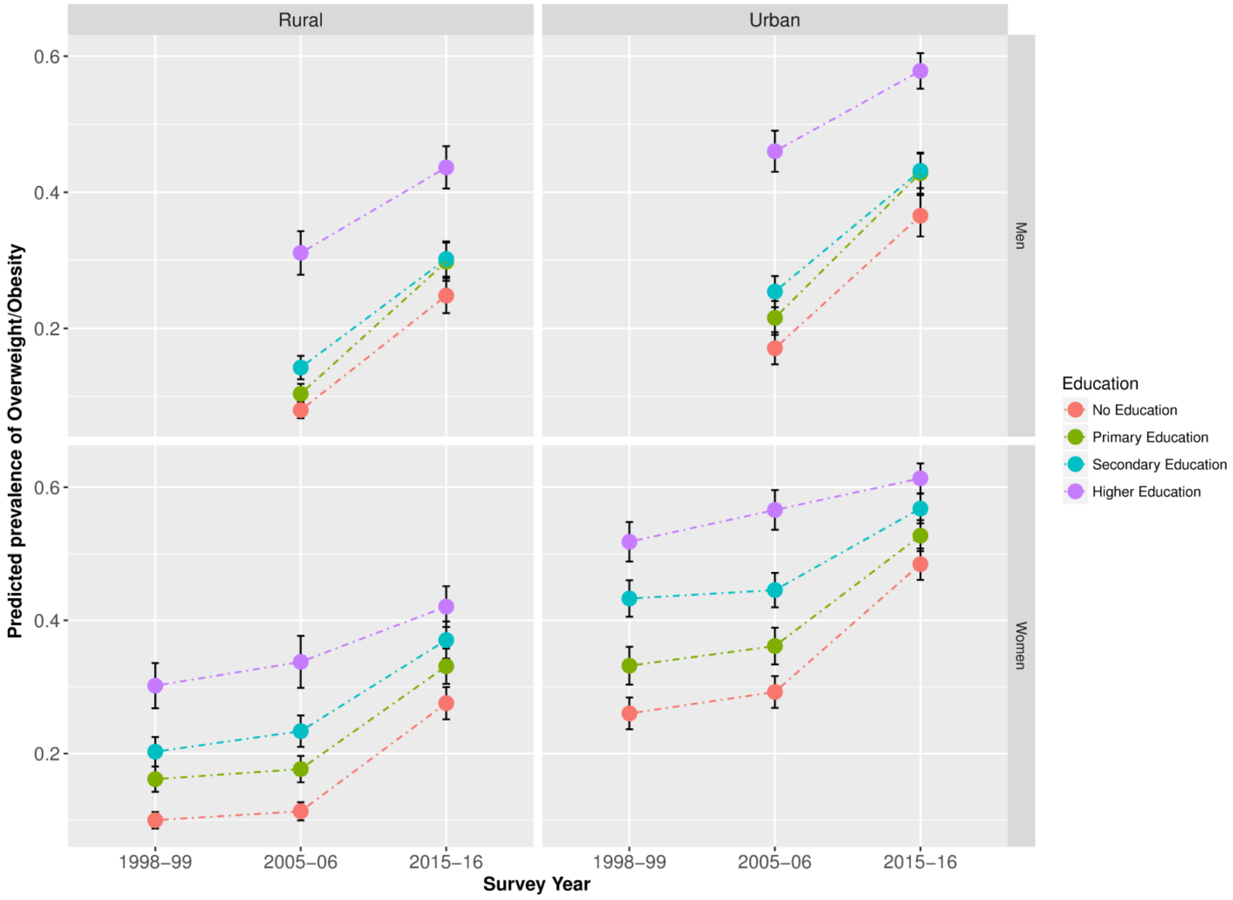

Figure A2. Predicted prevalence* of overweight/obesity in India by Standard of Living (using South Asian BMI cut-offs) (1998-2016)

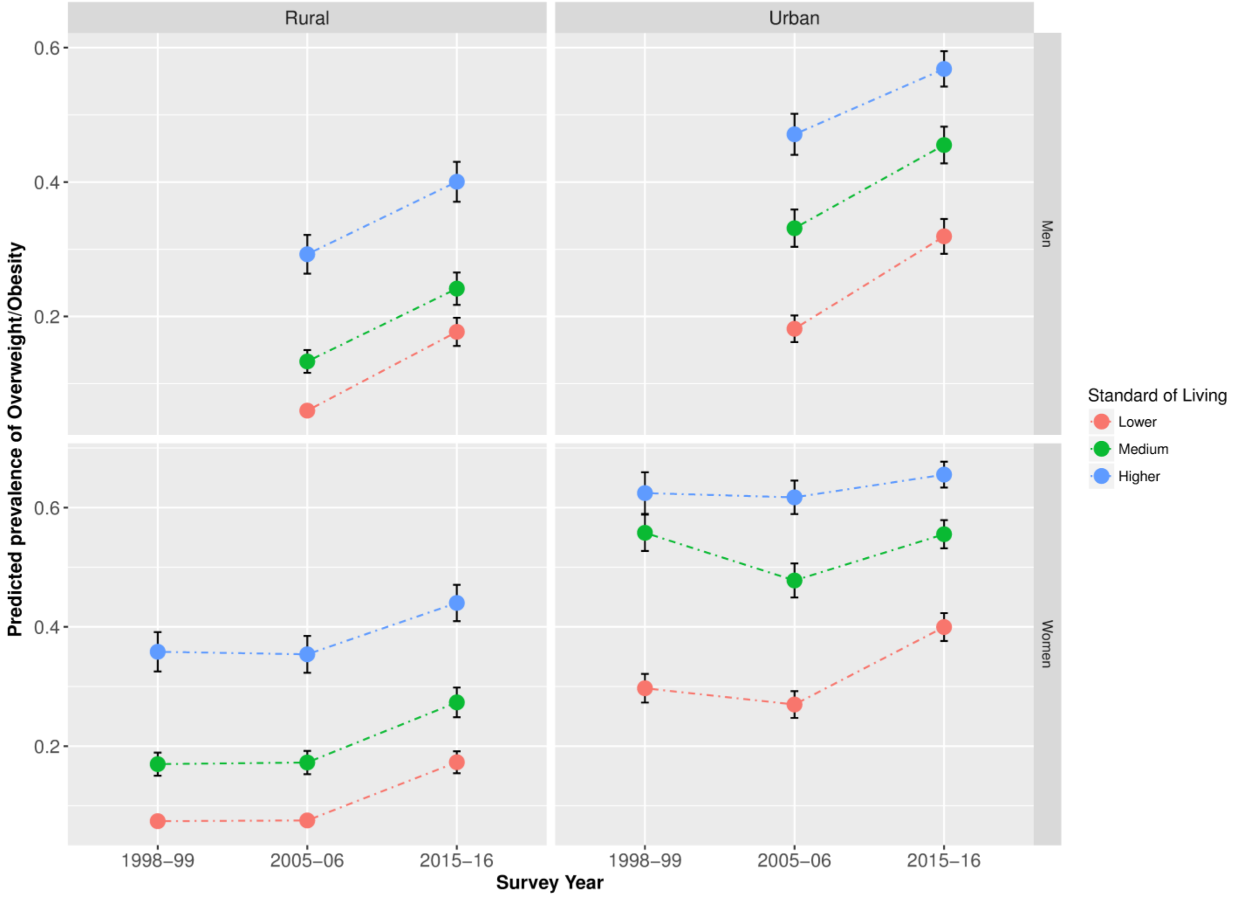

Supplement: Supplementary file 1 [file bmjopen-2018-023935supp001.pdf]
