# Supplementary material for: Trends in the socioeconomic patterning of overweight/obesity in India: a repeated cross-sectional study using nationally representative data
Source: BMJ Open. 2018 Oct 21;8(10):e023935. doi: 10.1136/bmjopen-2018-023935 (PMC6196932; doi:10.1136/bmjopen-2018-023935)
Supplement: Author's manuscript [file bmjopen-2018-023935.draft_revisions.pdf]

BMJ Open

BMJ Open is committed to open peer review. As part of this commitment we make the peer review history of every article we publish publicly available.

When an article is published we post the peer reviewers' comments and the authors' responses online. We also post the versions of the paper that were used during peer review. These are the versions that the peer review comments apply to.

The versions of the paper that follow are the versions that were submitted during the peer review process. They are not the versions of record or the final published versions. They should not be cited or distributed as the published version of this manuscript.

BMJ Open is an open access journal and the full, final, typeset and author-corrected version of record of the manuscript is available on our site with no access controls, subscription charges or pay-per-view fees (http://bmjopen.bmj.com).

If you have any questions on BMJ Open's open peer review process please email info.bmjopen@bmj.com

BMJ Open

Trends in the socioeconomic patterning of overweight/obesity in India: a repeated cross-sectional study using nationally representative data

Journal:	*BMJ Open*
Manuscript ID	bmjopen-2018-023935
Article Type:	Research
Date Submitted by the Author:	03-May-2018

Complete List of Authors:	Luhar, Shammi; London School of Hygiene and Tropical Medicine Faculty of Epidemiology and Population Health, Population Health<br>Mallinson, Poppy; London School of Hygiene and Tropical Medicine<br>Clarke, Lynda; London School of Hygiene and Tropical Medicine<br>Kinra, Sanjay; London School of Hygiene And Tropical Medicine

Keywords:	Overweight, Obesity, Socioeconomic position, Urban India, Rural India, Multilevel logistic regression

SCHOLARONE™
Manuscripts

For peer review only

Trends in the socioeconomic patterning of overweight/obesity in India: a repeated cross-sectional study using nationally representative data

Authors:

Shammi Luhar (shammi.luhar@lshtm.ac.uk) Department of Population Health, London School of Hygiene and Tropical Medicine

Poppy Mallinson (poppy.mallinson1@lshtm.ac.uk) Department of Non-Communicable Disease Epidemiology, London School of Hygiene and Tropical Medicine

Lynda Clarke (lynda.clarke@lshtm.ac.uk) Department of Population Health, London School of Hygiene and Tropical Medicine

Sanjay Kinra (sanjay.kinra@lshtm.ac.uk) Department of Non-Communicable Disease Epidemiology, London School of Hygiene and Tropical Medicine

Corresponding author

Shammi Luhar,

Department of Population Health,

London School of Hygiene and Tropical Medicine,

Keppel Street,

London, UK

WC1E 7HT

email: shammi.luhar@lshtm.ac.uk

Telephone: +447495993486

Word Count

2865

For peer review only

Abstract

Objectives

We aimed to examine trends in prevalence of overweight/obesity among adults in India by socioeconomic position (SEP) between 1998 and 2016.

Design

Repeated cross-sectional study using nationally representative data from the 1998/1999, 2005/2006 and 2015/16 nationally representative National Family Health Surveys of India. Multilevel logistic regression was used to assess trends in prevalence of overweight/obesity by SEP.

Setting

26, 29 and 36 Indian states or union territories, in 1998/99, 2005/06 and 2015/16, respectively.

Participants

628,795 ever-married women aged 15–49 years and 93,618 ever-married men aged 15-54.

Primary outcome measure

Overweight/obesity defined by body mass index >24.99 kg/m^2 (not overweight/obese reference category: body mass index <25.00 kg/m^2).

Results

Between 1998 and 2016, overweight/obesity prevalence increased in both urban and rural areas. In all periods, overweight/obesity prevalence was higher among higher SEP individuals (measured by educational attainment and a standard of living index), compared lower SEP individuals. Trends in the socioeconomic patterning of overweight/obesity differed slightly between urban and rural areas. In urban areas, overweight/obesity prevalence increased over the study period among lower SEP men and women, whereas no notable changes were identified among higher SEP women. Among higher SEP men, we identified some increase in the prevalence of overweight/obesity, however, the increase among lower SEP men was greater. In rural areas, more similar increases in the prevalence of overweight/obesity were found among all individuals across the study period, irrespective of SEP.

Conclusions

We identified some convergence of overweight/obesity prevalence across SEP in urban areas among both men and women, with fewer signs of convergence across SEP groups in rural areas. Efforts are therefore needed to slow the increasing trend among poorer Indians.

Keywords

Overweight, obesity, socioeconomic position, Urban India, Rural India, Multilevel logistic regression

For peer review only

For peer review only - http://bmjopen.bmj.com/site/about/guidelines.xhtml

Strengths and Limitations of this study

Our use of the most recent nationally representative data available for Indian adults make our results the most up-to-date estimates of the socioeconomic patterning of overweight/obesity, and their trends, in India.

Using a large nationally representative data set also enabled us to generate both precise and nationally generalisable overweight/obesity prevalence trends.

Body Mass Index was the only measure used to define overweight/obesity, and prevalence estimates may vary based on the adiposity measure used and the cut-offs used. However, we would not expect the reported socioeconomic patterning of overweight/obesity, and trends, to change considerably between measures.

Our results may mask subnational variation in overweight/obesity prevalence and trends, especially given large subnational differences in economic growth, demography and culture between India's states.

Introduction

Overweight and obesity present considerable challenges to the maintenance of global health improvements due to its association with many non-communicable diseases (NCDs) [1]. The World Health Organization's (WHO) aim to reduce global obesity to 2010 levels by 2025[1], is threatened by the increasing prevalence of over-nutrition in India[2], where nearly a sixth of the global population lives[3].

In India, economic growth and rising incomes have been accompanied by increases in the proportion of Indians classified as overweight or obese. The prevalence of overweight/obesity more than doubled for adult women from 9% to 21% between 1998 and 2016, while increasing from 11% to 19% among adult men between 2005 and 2016[245]. At the same time, undernutrition and infectious diseases continue to threaten population health[6789], presenting dilemmas about the appropriate allocation of scarce public finances and policy attention.

In low-income countries, overweight and obesity is usually more prevalent among higher socioeconomic position (SEP) groups[210111213], whereas the opposite is observed in most high-income countries, where lower SEP individuals are more likely to be overweight or obese[1013]. Although considered a lower middle-income country[14], India has experienced considerable economic growth between 1998 and 2015[15], and how this has impacted the prevalence of overnutrition by SEP is unknown.

In this study, we aim to estimate recent trends in overweight/obesity by SEP in India. Our results are intended to inform health policy decisions by identifying groups currently most at risk of overweight/obesity, and those that have experienced the largest increases in prevalence between 1998 and 2016[16]. We hypothesise that between 1998 and 2016, the prevalence of overweight/obesity increased in all SEP groups, in both urban and rural areas, however, with greater increases among lower SEP individuals than higher SEP individuals.

For peer review only - http://bmjopen.bmj.com/site/about/guidelines.xhtml

Methods

Study Population

The National Family Health Survey (NFHS) Waves 2, 3 and 4, collected in 1998-99, 2005-06 and 2015-16, respectively, gathered health and demographic data on 89,199, 124,385 and 699,686 eligible women in waves 2, 3 and 4, respectively, in addition to 74,369 and 112,122 eligible men in waves 3 and 4, respectively[245]. As NFHS-2 only collected data on ever-married women, we restricted the sample across survey waves to this population, to allow comparability over time. Pregnant women were not included in our analysis as their pregnancy may bias their assessment of weight status. In each of the waves, multi-stage sampling approaches were adopted, and sampling weights were provided in the data sets[245].

Outcome

In each survey, the participants' height and weight were measured and used to calculate Body Mass Index (BMI). As individuals with a BMI over 24.99kg/m^2 are found to be at higher risk of NCDs and mortality[1718], we categorised individuals as either overweight/obese (BMI over 24.99kg/m^2), or not overweight/obese (BMI less than or equal to 24.99kg/m^2), based on the WHO definition[1]. We additionally used cut-off values recommended for use among Asian populations to verify the trends we initially identified[19], whereby individuals with a BMI greater than 22.99kg/m^2 were classified as overweight/obese. Lower BMI cut-off values may be more appropriate among Asian populations, given a potentially higher risk of overweight/obesity related diseases at lower BMI levels compared to populations upon which initial classifications were based[19].

Independent Variables

The independent variables we used in the study were an index of standard of living, educational attainment, age, and marital status.

We allocated individuals in all the surveys to one of the following four education categories, based on the number of years of schooling: None (0 years); primary (1-5 years); secondary (6-12 years); higher (12+ years). We used Education as a measure of SEP as it may indicate employable skills that expose individuals to more opportunities to earn higher incomes.

The NFHS contains a wealth index, constructed using Principal Components Analysis (PCA) in each wave separately, using information on household asset ownership and household characteristics. As the original wealth index cannot be appropriately compared over time, and as we intended to stratify our analysis by urban and rural areas, we constructed a new index, as an alternative measure of SEP, using PCA from 26 assets and characteristics available in all the waves[245]. Based on our new wealth scores derived from weightings given to each asset or characteristic, households were classified as either 'lower', 'medium' or 'higher' standard of living (SoL). Asset-based indices are commonly used in cross sectional studies conducted in low and middle-income countries, where income data may be an unreliable indicator of overall SEP, particularly in rural

For peer review only - http://bmjopen.bmj.com/site/about/guidelines.xhtml

For peer review only

areas[20]. For instance, households may receive income from a variety of sources, which may be difficult to recall, or income may be received in kind[2021] rather than monetarily. Consequently, a household's stock of assets may provide a more reliable measure of current SEP[20].

We adjusted our final models for the respondent's age (categorised as 15-29; 30-39, and 40-49 (40-54) for women (men)), as it has been reported in previous studies that overweight/obesity prevalence increases with age[22]. Additionally, older adults may have accumulated more assets over a longer lifespan, potentially, confounding the association between SEP and overweight/obesity. Marital status (categorised as 'currently married', or 'not currently married') was introduced as a covariate as it has been found to be positively associated with overweight and obesity in India[11] and could confound the reported association between SEP and overweight/obesity.

Statistical Analysis

We initially calculated the prevalence of overweight/obesity in each standard of living index and educational attainment category, by sex and urban/rural residence. We accounted for the complex survey design of the data using sampling weights. Separately for urban and rural areas, we calculated the ratio of the prevalence between the highest and lowest socio-economic status group of our two main SEP variables (eg. higher to lower standard of living, and higher to no education) in each of the survey waves. Additionally, we calculated the percentage change in the prevalence of overweight/obesity by each category of standard of living and educational attainment.

Separately for urban and rural areas, and sex, we fitted multilevel logistic regression models with random intercepts for primary sampling units and states. We modelled the log odds ratio of overweight/obesity in each category of the SEP variable of interest in each of the survey waves by fitting a survey specific interaction term. The regression models were adjusted for the covariates mentioned in the independent variables section, in addition to the remaining SEP variable. No evidence of multicollinearity of independent variables with the main exposure of interest was detected when examining changes in the standard error once new variables were added. Finally, we derived and reported the predicted prevalence of overweight/obesity from the model, in addition to their 95% confidence bounds. Adjusted analyses were also carried out using Asian specific BMI cut-offs to observe if the trends identified varied depending on the outcome measure used.

Patient and public involvement

Publicly available survey data was used for the analysis and no patients were involved in the study.

Results

The study population generally experienced increasing educational attainment and standard of living over the period of analysis. For instance, whereas the percentage of women in the sample classified as having no

For peer review only - http://bmjopen.bmj.com/site/about/guidelines.xhtml

education declined over the study period, and did not change notably for men, the percentage with secondary education in the 2015-16 survey was higher than in previous ones. Additionally, in both rural and urban areas, the percentage of individuals from lower SoL households declined, whilst the percentage from higher SoL households increased between 1998 and 2016 (Table 1).

Table 1. Characteristics of study participants across NFHS waves with recorded BMI information

						Women				Men
	NFHS 2 (1998-99)		NFHS 3 (2005-06)		NFHS 4 (2015-16)		NFHS 3 (2005-06)		NFHS 4 (2015-16)	
	Freq.	Prop. *	Freq.	Prop. *	Freq.	Prop. *	Freq.	Prop. *	Freq.	Prop. *
Normal weight	68069	0.88	68433	0.81	377177	0.77	60973	0.88	89418	0.82
Overweight/obese	9544	0.12	15720	0.19	110567	0.23	8236	0.12	19282	0.18
Age 15-29	32838	0.42	31680	0.38	168689	0.35	33971	0.49	50170	0.46
Age 30-39	26741	0.34	30846	0.37	174552	0.36	17603	0.25	27707	0.25
Age 40-49 (40-54 males)	18034	0.23	21627	0.26	144503	0.30	17635	0.25	30823	0.28
No Education	38217	0.49	33362	0.40	175180	0.36	9920	0.14	14593	0.13
Primary	13494	0.17	13376	0.16	72470	0.15	10763	0.16	13952	0.13
Secondary	18785	0.24	30527	0.36	199305	0.41	38101	0.55	63300	0.58
Higher	7097	0.09	6881	0.08	40789	0.08	10398	0.15	16855	0.16
Low SoL (Rural)	28408	0.54	21262	0.44	64998	0.19	14615	0.42	11842	0.17
Middle SoL (Rural)	18616	0.35	15929	0.33	120050	0.36	12508	0.36	25338	0.35
High SoL (Rural)	5869	0.11	10645	0.22	149191	0.45	7409	0.21	34202	0.48
Low SoL (Urban)	16444	0.67	17263	0.48	33609	0.25	17329	0.50	8773	0.27
Middle SoL (Urban)	5682	0.23	10147	0.28	50027	0.38	9613	0.28	11925	0.36
High SoL (Urban)	2310	0.09	8832	0.24	49540	0.37	7694	0.22	12389	0.37
Married	72605	0.94	78608	0.93	460162	0.94	42008	0.61	68323	0.63
Not married	5008	0.06	5545	0.07	27582	0.06	27201	0.39	40377	0.37

The prevalence of overweight/obesity increased in each successive survey wave for both of our samples of men and women. In rural India, the prevalence among men almost tripled from 0.059 to 0.148 between 2005 and 2016, and among women, the prevalence increased from 0.059 to 0.182 between 1998 and 2016. In urban India, the prevalence among women increased to 0.385 in 2015-16, from 0.236 in 1998-99, whereas the prevalence among urban men increased from 0.167 to 0.276 between 2005 and 2016 (Figure 1).

Figure 1. Prevalence (weighted) of overweight/obesity in urban and rural India, among men and women

[FIGURE 1 ABOUT HERE]

In all survey waves, and for men and women in both urban and rural areas, the prevalence of overweight/obesity was highest among participants with higher education and from a higher SoL, whereas the lowest prevalence of overweight/obesity was found among participants with no education and from a lower SoL.

However, over the study periods for both men and women, the greatest percentage increase in overweight/obesity prevalence was observed among participants from the lowest SoL category and participants with no education. Consequently, the ratio of the prevalence of overweight/obesity in all of the highest, compared to the lowest, SEP groups, reduced over time (Table 2 and 3).

Table 2. Percentage of respondents classified as overweight/obese, by Education level (1998-2016)

					Women			Men
		1998-99	2005-06	2015-16	% change	2005-06	2015-16	% change
		%	%	%	1998-2016	%	%	2005-2016
Rural	Education**							
	No Education	3.38	5.26	13.91	311.54	3.05	10.79	253.77
	Primary	7.93	10.01	18.45	132.66	4.22	14.06	233.18
	Secondary	10.8	14.19	21.82	102.04	6.57	14.56	121.61
	Higher	15.85	22.79	26.73	68.64	15.32	22.32	45.69
	Ratio*	4.69	4.33	1.92		5.02	2.07	
Urban	Education**							
	No Education	13.53	18.49	32.17	137.77	7.73	18.28	136.48
	Primary	19.45	24.45	37.21	91.31	10.9	23.86	118.90
	Secondary	27.18	33.04	40.15	47.72	15.24	26.33	72.77
	Higher	35.35	41.79	41.56	17.57	28.39	34.87	22.82
	Ratio*	2.61	2.26	1.29		3.67	1.91	

*Ratio of the percentage among individuals with Higher education and no education
** Chi2 test p-value of each strata's association with overweight/obesity p<0.001

Table 3. Percentage of respondents classified as overweight/obese, by Standard of Living (1998-2016)

					Women			Men
		1998-99	2005-06	2015-16	% change	2005-06	2015-16	% change
		%	%	%	1998-2016	%	%	2005-2016
Rural	Standard of Living**							
	Lower SoL	2.35	3.01	6.65	182.98	1.79	4.96	177.09
	Middle SoL	8.22	8.88	12.94	57.42	5.66	9.47	67.31
	Higher SoL	22.93	25.15	27.74	20.98	17.49	22.3	27.50
	Ratio*	9.76	8.36	4.17		9.77	4.50	
Urban	Standard of Living**							
	Lower SoL	16.32	17.36	24.91	52.63	8.92	16.01	79.48
	Middle SoL	39.11	35.01	38.83	-0.72	20.61	26.89	30.47
	Higher SoL	46.93	48.4	46.87	-0.13	30.59	35.77	16.93
	Ratio*	2.88	2.79	1.88		3.43	2.23	

For peer review only

*Ratio of the percentage in the highest and lowest socio-economic group
** Chi2 test p-value of each strata's association with overweight/obesity p<0.001

Figure 2. Predicted prevalence* of overweight/obesity in India, by Educational attainment (1998-2016)

[FIGURE 2 ABOUT HERE]

Figure 3. Predicted prevalence* of overweight/obesity in India, by Standard of Living (1998-2016)

[FIGURE 3 ABOUT HERE]

After adjusting for marital status and age, in urban areas, the predicted prevalence of overweight/obesity among lower SEP women increased over the study period for both men and women, whereas no notable changes were observed among higher SEP women. Among urban men, we observed some increase in the prevalence of overweight/obesity among high SEP respondents, however, the increase among low SEP men was greater. Among both rural men and women, more similar increases were observed among individuals from all SEP groups over the study period (Figures 2 and 3). Equivalent trends were found when using the BMI cut-offs recommended for Asian populations (Figures A1 and A2).

Discussion

We found that, although overweight/obesity prevalence increased with SEP, in urban areas no notable change in the prevalence of overweight/obesity was observed among higher SEP women, whereas the prevalence among lower SEP women increased considerably between 1998-2016. The prevalence increase of overweight/obesity was greater among lower SEP urban men compared with higher SEP counterparts between 2005 and 2016. Consequently, some convergence of overweight/obesity across SEP was observed in urban areas among both men and women. In rural areas however, overweight/obesity prevalence increased similarly among individuals in all SEP groups, with fewer signs of convergence across SEP groups yet.

Strengths and limitations

The main strength of our study is our use of the most recent nationally representative data available for India, making our results the most up-to-date estimates of overweight/obesity trends by SEP.

Our study however has some limitations. Firstly, we derive our only measure of overweight/obesity from BMI, rather than complement our results with alternative measures of overweight/obesity, such as waist circumference[23,24] and body fat percentage. Consequently, the prevalence estimates we report may vary

For peer review only - http://bmjopen.bmj.com/site/about/guidelines.xhtml

depending on the adiposity measure and the exact definitions/cut-offs used. However, given the high correlation between BMI and measures including waist circumference among Indians[25], we would not expect the reported associations between overweight/obesity and SEP, and trends, to change considerably between measures.

Secondly, to ensure the population of sampled women was comparable over time, we limited our analysis to ever-married women, as this was the selection criteria in the NFHS-2 survey. Prevalence of overweight/obesity is generally higher among never-married women[26], for instance in the NFHS-4 survey data, the prevalence of overweight/obesity was 6.6% among never-married women, compared to 25.0% among currently married women. This may have lead us to overestimate overweight/obesity prevalence among women, as the weighted percentage of never-married women were 19.8% and 22.5% in the 2005-06 and 2015-16 samples, respectively. However, although individual point estimates may be affected, we do not expect the association between overweight/obesity and SEP we identified to be overestimated.

Our SoL index may also imperfectly capture household wealth. For instance, no indication about the quality of assets used in the measure were included, potentially misclassifying certain households[2027]. However, as three broad SoL groups across a large data set were defined, we do not expect any misclassification to substantially bias our results. Additionally, the association between the true SEP and certain assets included in the SoL index may differ between urban and rural areas. We attempted to account for differences in the value of certain assets by calculating separate indices for urban and rural areas, however, differences in the value of some assets may still exist within broad geographical areas, for instance between states.

Finally, our results may mask variation in subnational prevalence and trends, especially given subnational differences between states in economic growth, demography and culture. For instance, research in India has found that in states with a higher prevalence of overweight, lower and higher SEP group may show a converging risk of overweight/obesity, whereas divergent trends have been identified in states with the highest proportion of underweight individuals[28].

<u>Comparison with other research</u>

The only other India-specific national study we found on this topic did not identify any change in the overweight/obesity-SEP association between 1998-99 and 2005-06 in urban or rural India; with a persisting higher prevalence among high SEP groups[29]. Beyond 2005-06, the authors predicted that future overweight/obesity prevalence would show a similar social patterning as they expected future economic gains to almost solely benefit higher SEP individuals. By contrast, the converging socio-economic patterning of overweight/obesity we have identified in urban areas indicates that economic growth in the past decade may either have been more egalitarian than previously expected, the cost of high calorie food may have become less expensive, or even the pool of susceptible higher SEP individuals may be becoming saturated.

For peer review only - http://bmjopen.bmj.com/site/about/guidelines.xhtml

For peer review only

Converging overweight/obesity prevalence between higher and lower SEP groups has been identified sub-nationally in India, when restricted to states defined by a high overall prevalence of overweight[28], mirroring our finding in urban areas. This may suggest that convergence is restricted to areas that have moved beyond the earliest stages of the epidemiological transition.

Though not reported in previous nationally representative studies in India, a converging socioeconomic patterning of overweight/obesity has been noted in some other low and middle-income countries, where the highest increases in overweight prevalence have been found among women working in manual labour[30], among the lowest wealth and income groups[313233] and among rural residents[34].

<u>Potential mechanisms</u>

In rural areas we identified similar increases in prevalence among individuals from all SEP groups. Some studies suggest that in low-income settings, increases in overweight and obesity are restricted to higher SEP individuals, which may be due to changing dietary patterns towards fatty and sugary convenience foods[91011121335], however, the rising prevalence among lower SEP individuals indicates that they may also be increasingly exposed to high calorie foods. Some researchers have also suggested that this mechanism is stronger in low-income or rural settings due to more favourable perceptions of large body sizes across socioeconomic status[13363738].

In urban India, the greater increase in overweight/obesity prevalence among lower SEP individuals mirrors similar findings from places at relatively later stages of economic development, where some researchers have suggested that high prices of low calorie foods may price lower SEP individuals out of healthy diets[13394041]. Additionally, lower SEP individuals in urban areas may be more exposed to sedentary lifestyles driven by technological advances replacing manual energy-exerting labour, and improved transport links[4243]. Increased health consciousness, in combination with the ability to afford low calorie diets, may explain why no notable change in overweight/obesity prevalence among the higher SEP urban population was found[134445] in addition to the potential saturation of individuals susceptible to becoming overweight or obese.

<u>Implications</u>

Some studies argue that in India NCD risk factors are almost exclusively an issue for higher SEP individuals[46]. However, our finding that overweight/obesity prevalence has increased among lower SEP individuals in both urban and rural areas implies that to consider overweight/obesity as ‘diseases of affluence’[1047] may not be appropriate in India’s current context. Efforts to tackle the overall increasing overweight/obesity trend must be inclusive of both the urban and rural poor. This may be especially urgent due to the compounding effect of overweight/obesity and associated NCDs on infectious diseases, which are still highly prevalent among the poor.

For peer review only

Recent initiatives to raise population health include the launch of an integrated National Health Mission[48] which aims to address deficiencies in healthcare delivery across the socioeconomic spectrum in urban and rural areas. Such initiatives may benefit from information about the increasing prevalence among low SEP Indians, as future action aimed at preventing overweight and obesity can be targeted accordingly. Due to the positive association of overweight and obesity with non-communicable diseases such as stroke and diabetes[4950] urgency is required in addressing this modifiable risk factor especially as it could compound existing health complications among poorer Indians, where communicable disease and under-nutrition related diseases already tend to be more prevalent.

Conclusion

Although India is still considered as a lower middle-income country, we have identified some convergence of overweight/obesity prevalence across SEP in urban areas among both men and women, with fewer signs of convergence across SEP groups in rural areas. Our findings suggest that an urgent response is needed to slow the increasing trend among poorer Indians, particularly as increasing exposure to over-nutrition related diseases may compound an already high exposure to infectious diseases.

Conflicts of interest

The authors have no conflicts of interest to declare

Author Contributions

The authors' responsibilities were as follows: Shammi Luhar and Sanjay Kinra designed the study; Shammi Luhar performed the data analysis and takes responsibility for the final content; Shammi Luhar interpreted the results; Shammi Luhar drafted the manuscript; Poppy Mallinson, Lynda Clarke and Sanjay Kinra reviewed and approved the final manuscript.

Data statement

All datasets in this analysis are available at http://www.measuredhs.com.

Funding

This research received no specific grant from any funding agency in the public, commercial or not-for-profit sectors.

Ethical Approval

The analysis of secondary data was approved by the London School of Hygiene and Tropical Medicine's Research ethics committee.

Informed Consent

For peer review only

Data used was anonymised. After receiving detailed information on the survey, participants were asked to give consent to participate in the NFHS surveys by signing a consent declaration.

Acknowledgements

We thank measure DHS for granting us access to the data used in this study.

References

[1] World Health Organization, 2016. Fact Sheet 311: obesity and overweight. Available at: http://www.who.int/mediacentre/factsheets/fs311/en/ [Accessed 13 Jul. 2017].
[2] International Institute for Population Sciences (IIPS) and ORC Macro 2017. National Family Health Survey (NFHS-4), 2014-15: India. Mumbai: IIPS.
[3] DeSA, U.N., 2013. World population prospects: the 2012 revision. Population division of the department of economic and social affairs of the United Nations Secretariat, New York.
[4] International Institute for Population Sciences (IIPS) and Macro International. 2007. National Family Health Survey (NFHS-3), 2005-06: India: Volume I. Mumbai: IIPS.
[5] International Institute for Population Sciences (IIPS) and ORC Macro 2000. National Family Health Survey (NFHS-2), 1998-99: India. Mumbai: IIPS.
[6] Wang, Y., Chen, H.J., Shaikh, S. and Mathur, P., 2009. Is obesity becoming a public health problem in India? Examine the shift from under-to overnutrition problems over time. Obesity Reviews, 10(4), pp.456-474.
[7] Yadav, S. and Arokiasamy, P., 2014. Understanding epidemiological transition in India. Global health action, 7(1), p.23248.
[8] Reid, C.M. and Thrift, A.G., 2005. Hypertension 2020: confronting tomorrow's problem today. Clinical and experimental pharmacology and physiology, 32(5-6), pp.374-376.
[9] Samal, S., Panigrahi, P. and Dutta, A., 2015. Social epidemiology of excess weight and central adiposity in older Indians: analysis of Study on global AGEing and adult health (SAGE). BMJ open, 5(11), p.e008608.
[10] Monteiro, C.A., Moura, E.C., Conde, W.L. and Popkin, B.M., 2004. Socioeconomic status and obesity in adult populations of developing countries: a review. Bulletin of the World Health Organization, 82(12), pp.940-946.
[11] Gouda, J. and Prusty, R.K., 2014. Overweight and obesity among women by economic stratum in urban India. Journal of health, population, and nutrition, 32(1), p.79.
[12] Kain, J., Vio, F. and Albala, C., 2003. Obesity trends and determinant factors in Latin America. Cadernos de Saude Publica, 19, pp.S77-S86.
[13] Dinsa, G.D., Goryakin, Y., Fumagalli, E. and Suhrcke, M., 2012. Obesity and socioeconomic status in devel- oping countries: a systematic review. Obesity reviews, 13(11), pp.1067-1079.
[14] World Bank, n.d. India. India | Data. URL https://data.worldbank.org/country/india (accessed 4.27.18).
[15] International Monetary Fund., 2018. GDP, current prices. IMF DataMapper. URL http://www.imf.org/external/datamapper/NGDPD@WEO/OEMDC/ADVEC/WEOWORLD/IND (accessed 4.27.18).
[16] Traill, W.B., 2006. Trends towards overweight in lower-and middle-income countries: some causes and economic policy options. The double burden of malnutrition, p.305.
[17] Di Angelantonio, E., Bhupathiraju, S.N., Wormser, D., Gao, P., Kaptoge, S., de Gonzalez, A.B., Cairns, B.J., Huxley, R., Jackson, C.L., Joshy, G. and Lewington, S., 2016. Body-mass index and all-cause mortality: individual-participant-data meta-analysis of 239 prospective studies in four continents. The Lancet, 388(10046), pp.776-786.
[18] Chen, Y., Copeland, W.K., Vedanthan, R., Grant, E., Lee, J.E., Gu, D., Gupta, P.C., Ramadas, K., Inoue, M., Tsugane, S. and Tamakoshi, A., 2013. Association between body mass index and cardiovascular disease mortality in east Asians and south Asians: pooled analysis of prospective data from the Asia Cohort Consortium
[19] WHO, E.C., 2004. Appropriate body-mass index for Asian populations and its implications for policy and intervention strategies. Lancet (London, England), 363(9403), p.157.
[20] Vyas, S. and Kumaranayake, L., 2006. Constructing socio-economic status indices: how to use principal components analysis. Health policy and planning, 21(6), pp.459-468.
[21] McKenzie, D.J., 2003. Measure inequality with asset indicators. Bureau for Research and Economic Analysis of Development, Centre for International Development, BREAD.
[22] Subramanian, S.V., Kawachi, I. and Smith, G.D., 2007. Income inequality and the double burden of under-and overnutrition in India. Journal of Epidemiology & Community Health, 61(9), pp.802-809.
[23] Misra A, Vikram NK, Gupta R, Pandey RM ,Wasir JS,Gupta VP.Waist circumference cutoff points and action levels for Asian Indians for identification of abdominal obesity. International Journal of Obesity 2006;30:106–11.
[24] Subramanian, S.V. and Smith, G.D., 2006. Patterns, distribution, and determinants of under-and overnutrition: a population-based study of women in India. The American Journal of clinical nutrition, 84(3), pp.633-640.

For peer review only - http://bmjopen.bmj.com/site/about/guidelines.xhtml

[25] Kuper, H., Taylor, A., Krishna, K.V.R., Ben-Shlomo, Y., Gupta, R., Kulkarni, B., Prabhakaran, D., Smith, G.D., Wells, J., Ebrahim, S. and Kinra, S., 2014. Is vulnerability to cardiometabolic disease in Indians mediated by abdominal adiposity or higher body adiposity. BMC public health, 14(1), p.1239.

[26] Lipowicz, A., Gronkiewicz, S. and Malina, R.M., 2002. Body mass index, overweight and obesity in married and never married men and women in Poland. American Journal of Human Biology, 14(4), pp.468-475.

[27] Falkingham, J. and Namazie, C., 2002. Measuring health and poverty: a review of approaches to identifying the poor. London: DFID Health Systems Resource Centre, p.7.

[28] Sengupta, A., Angeli, F., Syamala, T.S., Dagnelie, P.C. and van Schayck, C.P., 2015. Overweight and obesity prevalence among Indian women by place of residence and socio-economic status: Contrasting patterns from 'underweight states' and 'overweight states' of India. Social Science & Medicine, 138, pp.161-169.

[29] Subramanian, S.V., Perkins, J.M. and Khan, K.T., 2009. Do burdens of underweight and overweight coexist among lower socioeconomic groups in India?–. The American journal of clinical nutrition, 90(2), pp.369-376.

[30] Lopez-Arana, S., Avendano, M., van Lenthe, F.J. and Burdorf, A., 2014. Trends in overweight among women differ by occupational class: results from 33 low-and middle-income countries in the period 1992–2009. International Journal of Obesity, 38(1), p.97.

[31] Jones-Smith, J.C., Gordon-Larsen, P., Siddiqi, A. and Popkin, B.M., 2012. Is the burden of overweight shifting to the poor across the globe? Time trends among women in 39 low-and middle-income countries (1991–2008). International journal of obesity, 36(8), p.1114.

[32] Monteiro, C.A., Conde, W.L. and Popkin, B.M., 2007. Income-specific trends in obesity in Brazil: 1975–2003. American journal of public health, 97(10), pp.1808-1812.

[33] Nguyen, M.D., Beresford, S.A. and Drewnowski, A., 2007. Trends in overweight by socio-economic status in Vietnam: 1992 to 2002. Public health nutrition, 10(2), pp.115-121.

[34] Wang, H., Du, S., Zhai, F. and Popkin, B.M., 2007. Trends in the distribution of body mass index among Chinese adults, aged 20–45 years (1989–2000). International Journal of Obesity, 31(2), p.272.

[35] Khandelwal, S. and Reddy, K.S., 2013. Eliciting a policy response for the rising epidemic of overweight-obesity in India. obesity reviews, 14(S2), pp.114-125.

[36] Rguibi, M. and Belahsen, R., 2006. Body size preferences and sociocultural influences on attitudes towards obesity among Moroccan Sahraoui women. Body Image, 3(4), pp.395-400.

[37] Fernald, L.C., 2009. Perception of body weight: a critical factor in understanding obesity in middle-income countries. Journal of Womens Health, 18(8), pp.1121-1122.

[38] Gulati S, Misra A, Colles S et al. 2013. Dietary intakes and familial correlates of overweight/obesity: a four cities study in India. Annals of Nutrition and Metabolism 62: 279–90.

[39] Temple, N.J., Steyn, N.P., Fourie, J. and De Villiers, A., 2011. Price and availability of healthy food: A study in rural South Africa. Nutrition, 27(1), pp.55-58.

[40] Ramachandran, P., 2006. The double burden of malnutrition in India. Rome: FAO Food and Nutrition Papers - Food and Agriculture Organization of the United Nations, pp.99-160.

[41] Uustialo, U., Pietinen, P. & Puska, P., 2002. Dietary transition in developing countries: challenges for chronic disease prevention. In WHO. Globalization, diets and non-communicable diseases. Geneva, WHO. 25.

[42] Singh, R.B., Pella, D., Mechirova, V., Kartikey, K., Demeester, F., Tomar, R.S., Beegom, R., Mehta, A.S., Gupta, S.B., De, A.K. and Neki, N.S., 2007. Prevalence of obesity, physical inactivity and undernutrition, a triple burden of diseases during transition in a developing economy. The Five City Study Group. Acta cardiologica, 62(2), p.119.

[43] Unnikrishnan, A.G., Kalra, S. and Garg, M.K., 2012. Preventing obesity in India: Weighing the options. Indian journal of endocrinology and metabolism, 16(1), p.4.

[44] Lallukka T., Laaksonen M., Rahkonen O., Roos E., Lahelma E. 2009. Multiple socio-economic circumstances and healthy food habits. Cent. Eur. J. Public Health 17, 55–63

[45] Kearney, J., 2010. Food consumption trends and drivers. Philosophical transactions of the royal society B: biological sciences, 365(1554), pp.2793-2807.

[46] Subramanian, S.V., Corsi, D.J., Subramanyam, M.A. and Davey Smith, G., 2013. Jumping the gun: the problematic discourse on socioeconomic status and cardiovascular health in India. International journal of epidemiology, 42(5), pp.1410-1426.

[47] Ezzati, M., Vander Hoorn, S., Lawes, C.M., Leach, R., James, W.P.T., Lopez, A.D., Rodgers, A. and Murray, C.J., 2005. Rethinking the "diseases of affluence" paradigm: global patterns of nutritional risks in relation to economic development. PLoS medicine, 2(5), p.e133.

[48] D'Silva, J., 2013. Can India pull off its ambitious National Health Mission?. BMJ: British Medical Journal (Online), 346.

[49] Kivimäki, M., Kuosma, E., Ferrie, J.E., Luukkonen, R., Nyberg, S.T., Alfredsson, L., Batty, G.D., Brunner, E.J., Fransson, E., Goldberg, M. and Knutsson, A., 2017. Overweight, obesity, and risk of cardiometabolic multimorbidity: pooled analysis of individual-level data for 120 813 adults from 16 cohort studies from the USA and Europe. The Lancet Public Health, 2(6), pp.e277-e285.

[50] Babu, G.R., Murthy, G.V.S., Ana, Y., Patel, P., Deepa, R., Neelon, S.E.B., Kinra, S. and Reddy, K.S., 2018. Association of obesity with hypertension and type 2 diabetes mellitus in India: A meta-analysis of observational studies. World Journal of Diabetes, 9(1), p.40.

For peer review only

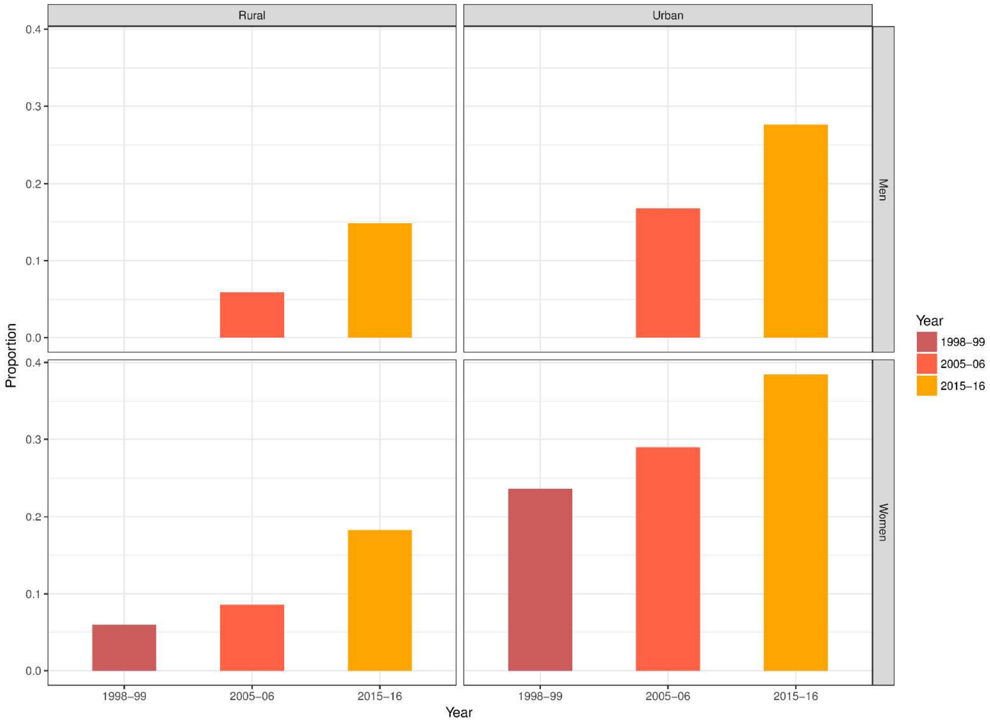

For peer review only

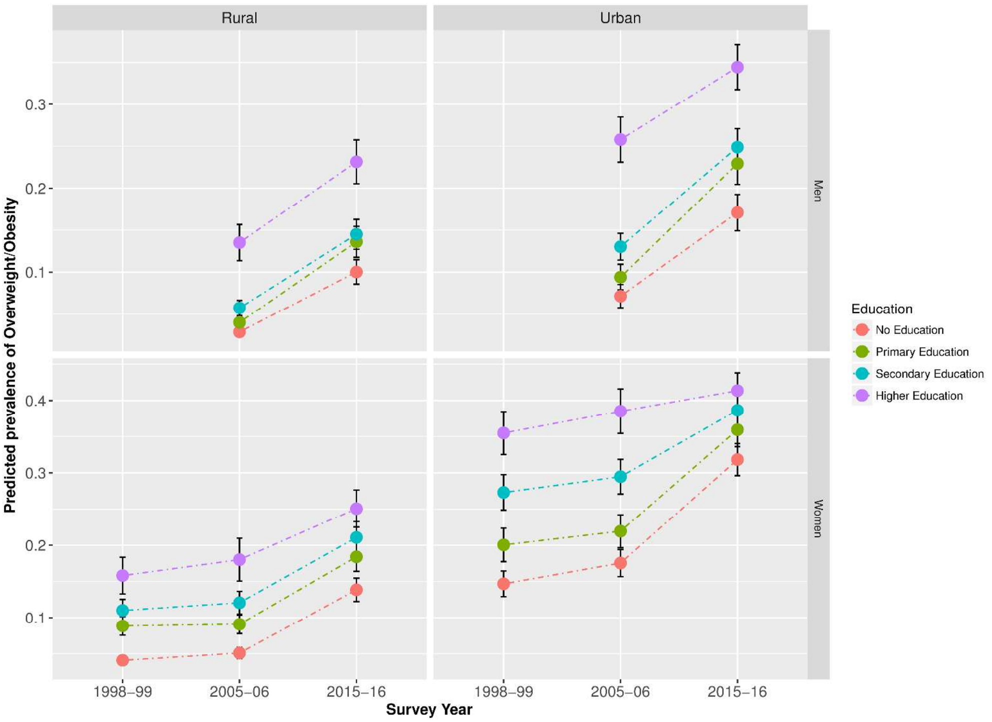

*Predicted prevalences and confidence intervals are based on multivariate regressions, and the models adjust for the respondent's age, current marital status and the socio-economic variable not considered as the main exposure.

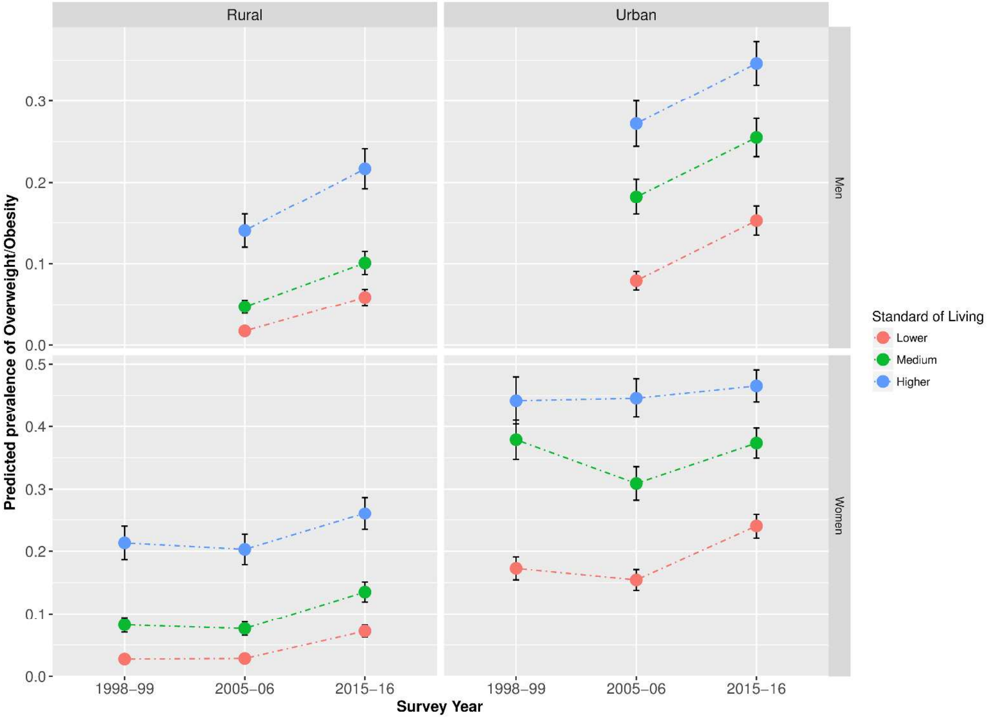

*Predicted prevalences and confidence intervals are based on multivariate regressions, and the models adjust for the respondent's age, current marital status and the socio-economic variable not considered as the main exposure.

For peer review only - http://bmjopen.bmj.com/site/about/guidelines.xhtml

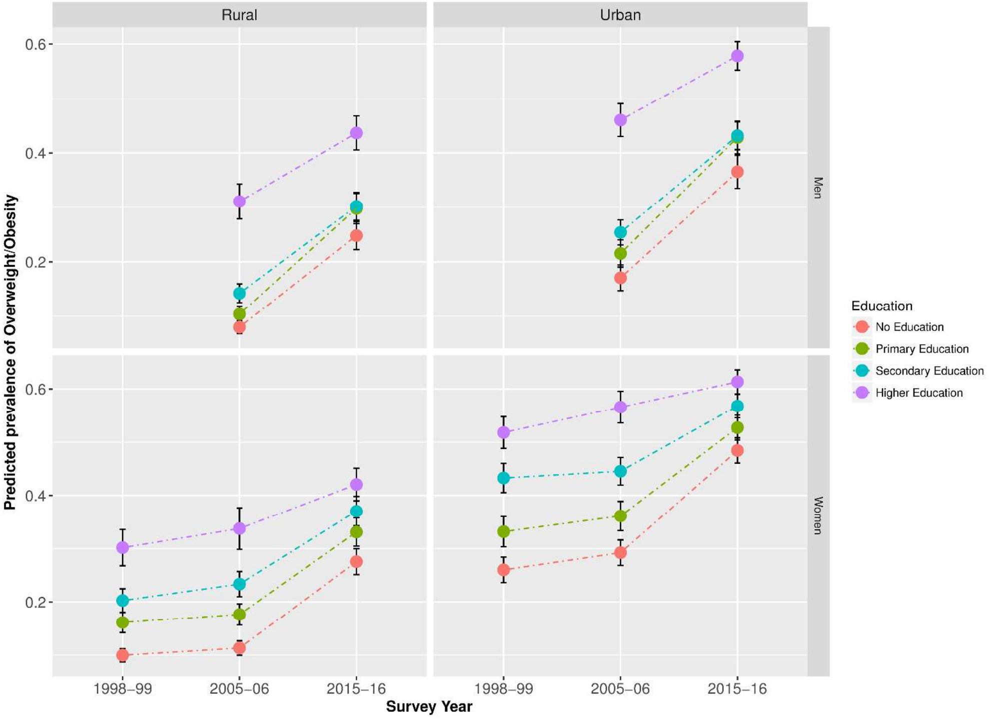

*Predicted prevalences and confidence intervals are based on multivariate regressions, and the models adjust for the respondent's age, current marital status and the socio-economic variable not considered as the main exposure.

For peer review only - http://bmjopen.bmj.com/site/about/guidelines.xhtml

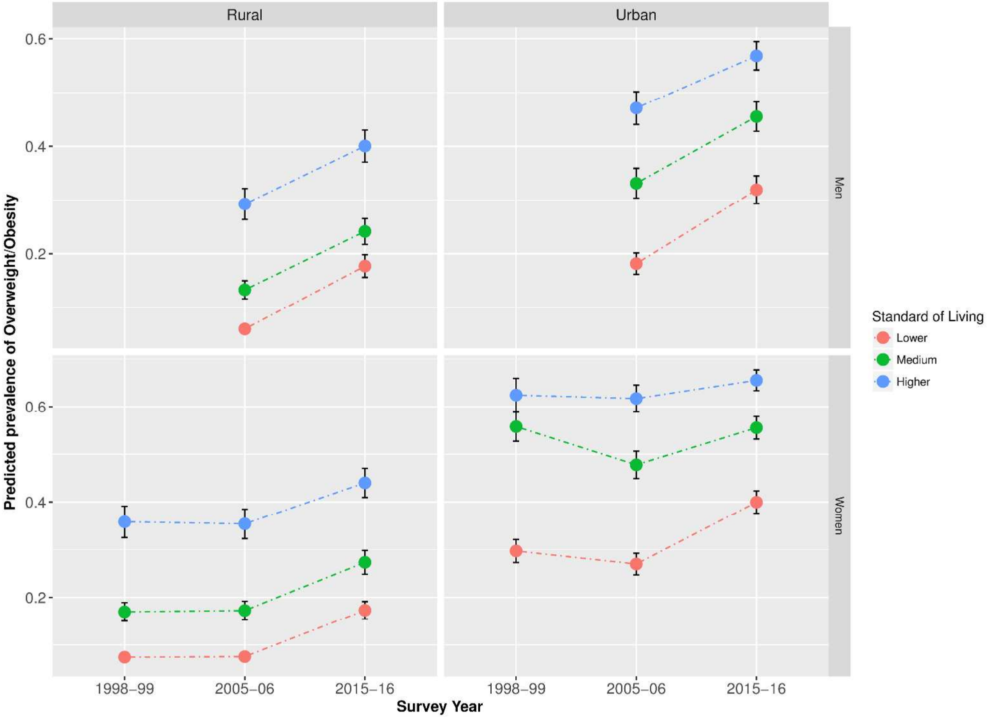

*Predicted prevalences and confidence intervals are based on multivariate regressions, and the models adjust for the respondent's age, current marital status and the socio-economic variable not considered as the main exposure.

For peer review only - http://bmjopen.bmj.com/site/about/guidelines.xhtml

Appendix A

Figure A1. Predicted prevalence* of overweight/obesity in India by Education (using South Asian BMI cut-offs) (1998-2016)

[FIGURE A1 ABOUT HERE]

Figure A2. Predicted prevalence* of overweight/obesity in India by Standard of Living (using South Asian BMI cut-offs) (1998-2016)

[FIGURE A2. ABOUT HERE]

For peer review only

Reporting checklist for cross sectional study.

Based on the STROBE cross sectional guidelines.

Instructions to authors

Complete this checklist by entering the page numbers from your manuscript where readers will find each of the items listed below.

Your article may not currently address all the items on the checklist. Please modify your text to include the missing information. If you are certain that an item does not apply, please write "n/a" and provide a short explanation.

Upload your completed checklist as an extra file when you submit to a journal.

In your methods section, say that you used the STROBE cross sectional reporting guidelines, and cite them as:

von Elm E, Altman DG, Egger M, Pocock SJ, Gotzsche PC, Vandenbroucke JP. The Strengthening the Reporting of Observational Studies in Epidemiology (STROBE) Statement: guidelines for reporting observational studies.

		Reporting Item	Page Number
Title	#1a	Indicate the study’s design with a commonly used term in the title or the abstract	1
Abstract	#1b	Provide in the abstract an informative and balanced summary of what was done and what was found	2
Background / rationale	#2	Explain the scientific background and rationale for the investigation being reported	3
Objectives	#3	State specific objectives, including any prespecified hypotheses	3
Study design	#4	Present key elements of study design early in the paper	4
Setting	#5	Describe the setting, locations, and relevant dates, including periods of recruitment, exposure, follow-up, and data collection	4
Eligibility criteria	#6a	Give the eligibility criteria, and the sources and methods of selection of participants.	4

For peer review only

	#7	Clearly define all outcomes, exposures, predictors, potential confounders, and effect modifiers. Give diagnostic criteria, if applicable	4
Data sources / measurement	#8	For each variable of interest give sources of data and details of methods of assessment (measurement). Describe comparability of assessment methods if there is more than one group. Give information separately for for exposed and unexposed groups if applicable.	4
Bias	#9	Describe any efforts to address potential sources of bias	4
Study size	#10	Explain how the study size was arrived at	4
Quantitative variables	#11	Explain how quantitative variables were handled in the analyses. If applicable, describe which groupings were chosen, and why	4
Statistical methods	#12a	Describe all statistical methods, including those used to control for confounding	5
	#12b	Describe any methods used to examine subgroups and interactions	5
	#12c	Explain how missing data were addressed	4
	#12d	If applicable, describe analytical methods taking account of sampling strategy	5
	#12e	Describe any sensitivity analyses	5
Participants	#13a	Report numbers of individuals at each stage of study—eg numbers potentially eligible, examined for eligibility, confirmed eligible, included in the study, completing follow-up, and analysed. Give information separately for for exposed and unexposed groups if applicable.	4
	#13b	Give reasons for non-participation at each stage	4
	#13c	Consider use of a flow diagram	4
Descriptive data	#14a	Give characteristics of study participants (eg demographic, clinical, social) and information on exposures and potential confounders. Give information separately for exposed and unexposed groups if applicable.	6

For peer review only

	#14b	Indicate number of participants with missing data for each variable of interest	4
Outcome data	#15	Report numbers of outcome events or summary measures. Give information separately for exposed and unexposed groups if applicable.	6
Main results	#16a	Give unadjusted estimates and, if applicable, confounder-adjusted estimates and their precision (eg, 95% confidence interval). Make clear which confounders were adjusted for and why they were included	7,8
	#16b	Report category boundaries when continuous variables were categorized	8
	#16c	If relevant, consider translating estimates of relative risk into absolute risk for a meaningful time period	8
Other analyses	#17	Report other analyses done—e.g., analyses of subgroups and interactions, and sensitivity analyses	7,8
Key results	#18	Summarise key results with reference to study objectives	8
Limitations	#19	Discuss limitations of the study, taking into account sources of potential bias or imprecision. Discuss both direction and magnitude of any potential bias.	8,9
Interpretation	#20	Give a cautious overall interpretation considering objectives, limitations, multiplicity of analyses, results from similar studies, and other relevant evidence.	9,10
Generalisability	#21	Discuss the generalisability (external validity) of the study results	9
Funding	#22	Give the source of funding and the role of the funders for the present study and, if applicable, for the original study on which the present article is based	11

The STROBE checklist is distributed under the terms of the Creative Commons Attribution License CC-BY. This checklist was completed on 01. May 2018 using http://www.goodreports.org/, a tool made by the EQUATOR Network in collaboration with Penelope.ai

For peer review only

BMJ Open

Trends in the socioeconomic patterning of overweight/obesity in India: a repeated cross-sectional study using nationally representative data

Journal:	*BMJ Open*
Manuscript ID	bmjopen-2018-023935.R1
Article Type:	Research
Date Submitted by the Author:	02-Aug-2018
Complete List of Authors:	Luhar, Shammi; London School of Hygiene and Tropical Medicine Faculty of Epidemiology and Population Health, Population Health<br>Mallinson, Poppy; London School of Hygiene and Tropical Medicine<br>Clarke, Lynda; London School of Hygiene and Tropical Medicine<br>Kinra, Sanjay; London School of Hygiene And Tropical Medicine
**Primary Subject Heading**:	Epidemiology
Secondary Subject Heading:	Nutrition and metabolism
Keywords:	Overweight, Obesity, Socioeconomic position, Urban India, Rural India, Multilevel logistic regression

SCHOLARONE™
Manuscripts

Trends in the socioeconomic patterning of overweight/obesity in India: a repeated cross-sectional study using nationally representative data

Authors:

Shammi Luhar (shammi.luhar@lshtm.ac.uk) Department of Population Health, London School of Hygiene and Tropical Medicine

Poppy Mallinson (poppy.mallinson1@lshtm.ac.uk) Department of Non-Communicable Disease Epidemiology, London School of Hygiene and Tropical Medicine

Lynda Clarke (lynda.clarke@lshtm.ac.uk) Department of Population Health, London School of Hygiene and Tropical Medicine

Sanjay Kinra (sanjay.kinra@lshtm.ac.uk) Department of Non-Communicable Disease Epidemiology, London School of Hygiene and Tropical Medicine

Corresponding author

Shammi Luhar,

Department of Population Health,

London School of Hygiene and Tropical Medicine,

Keppel Street,

London, UK

WC1E 7HT

email: shammi.luhar@lshtm.ac.uk

Telephone: +447495993486

Word Count

3025

For peer review only

Abstract

Objectives
We aimed to examine trends in prevalence of overweight/obesity among adults in India by socioeconomic position (SEP) between 1998 and 2016.

Design
Repeated cross-sectional study using nationally representative data from India collected in 1998/1999, 2005/2006 and 2015/16. Multilevel regressions were used to assess trends in prevalence of overweight/obesity by SEP.

Setting
26, 29 and 36 Indian states or union territories, in 1998/99, 2005/06 and 2015/16, respectively.

Participants
628,795 ever-married women aged 15–49 years and 93,618 men aged 15-54.

Primary outcome measure
Overweight/obesity defined by body mass index >24.99 kg/m^2.

Results
Between 1998 and 2016, overweight/obesity prevalence increased among men and women in both urban and rural areas. In all periods, overweight/obesity prevalence was consistently highest among higher SEP individuals. In urban areas, overweight/obesity prevalence increased considerably over the study period among lower SEP adults. For instance, between 1998 and 2016, overweight/obesity prevalence increased from approximately 15% to 32% among urban women with no education. Whereas the prevalence among urban men with higher education increased from 26% to 34% between 2005 and 2016, we did not observe any notable changes among high SEP urban women between 1998 and 2016. In rural areas, more similar increases in overweight/obesity prevalence were found among all individuals across the study period, irrespective of SEP. Among rural women with higher education, overweight/obesity increased from 16 to 25% between 1998 and 2016, whilst the prevalence among rural women with no education increased from 4% to 14%.

Conclusions
We identified some convergence of overweight/obesity prevalence across SEP in urban areas among both men and women, with fewer signs of convergence across SEP groups in rural areas. Efforts are therefore needed to slow the increasing trend of overweight/obesity among all Indians, as we found evidence suggesting it may no longer be considered a ‘diseases of affluence’.

Keywords
Overweight, obesity, socioeconomic position, Urban India, Rural India, Multilevel logistic regression

For peer review only - http://bmjopen.bmj.com/site/about/guidelines.xhtml

Strengths and Limitations of this study

- Our use of the most recent nationally representative data available for Indian adults make our results the most up-to-date estimates of the socioeconomic patterning of overweight/obesity, and their trends, in India.

- Using a large nationally representative data set also enabled us to generate both precise and nationally generalisable overweight/obesity prevalence trends.

- Body Mass Index was the only measure used to define overweight/obesity, and prevalence estimates may vary based on the adiposity measure used and the cut-offs used. However, we would not expect the reported socioeconomic patterning of overweight/obesity, and trends, to change considerably between measures.

- Our results may mask subnational variation in overweight/obesity prevalence and trends, especially given large subnational differences in economic growth, demography and culture between India's states.

Introduction

Overweight and obesity present considerable challenges to the maintenance of global health improvements due to its association with many non-communicable diseases (NCDs) [1]. The World Health Organization's (WHO) aim to reduce global obesity to 2010 levels by 2025[1], is threatened by the increasing prevalence of overweight and obesity in India[2], where nearly a sixth of the global population lives[3].

In India, economic growth and rising incomes have been accompanied by increases in the proportion of Indians classified as overweight or obese. The proportion of adult women classified as either overweight or more than doubled for adult women from 9% to 21% between 1998 and 2016, while increasing from 11% to 19% among adult men between 2005 and 2016[245]. At the same time, undernutrition and infectious diseases continue to threaten population health[6789], presenting dilemmas about the appropriate allocation of scarce public finances and policy attention.

In low-income countries, overweight and obesity is usually more prevalent among higher socioeconomic position (SEP) groups[210111213], whereas the opposite is observed in most high-income countries, where lower SEP individuals are more likely to be overweight or obese[1013]. Although considered a lower middle-income country[14], India has experienced considerable economic growth between 1998 and 2015[15], and how this has impacted the proportion classified as overweight or obese in different SEP groups is unknown.

In this study, we aim to estimate recent trends in the proportion of Indians considered overweight or obese by SEP in India. Our results are intended to inform health policy decisions by identifying groups currently most at risk of being overweight or obese, and those that have experienced the largest increases in prevalence between 1998 and 2016[16]. We hypothesise that between 1998 and 2016, the proportion classified as overweight or obese has increased in all SEP groups, in both urban and rural areas, however, with greater increases among lower SEP individuals than higher SEP individuals.

For peer review only - http://bmjopen.bmj.com/site/about/guidelines.xhtml

Methods

Study Population

The National Family Health Surveys (NFHS) 2, 3 and 4, collected in 1998-99, 2005-06 and 2015-16, respectively, gathered health and demographic data on 89,199, 124,385 and 699,686 eligible women in surveys 2, 3 and 4, respectively, in addition to 74,369 and 112,122 eligible men in surveys 3 and 4, respectively[245]. As NFHS-2 only collected data on ever-married women, we restricted the sample across surveys to this population, to allow comparability over time. Pregnant women were not included in our analysis as their pregnancy may bias their assessment of weight status. From this restricted sample, we further excluded women (1998-99: n=6182 (7.4%); 2005-06: n=3673 (4.2%); 2015-16: 7810 (1.6%)) and men (2005-06: n= 5160 (6.8%); 2015-16: n=3422 (3.1%)) with missing height and weight data. The analytic sample used in our main analysis consisted of 628,795 women aged 15–49 years and 93,618 men aged 15-54 across all three surveys, representing respondents with complete data across all the key variables. In each of the surveys, multi-stage sampling approaches were adopted, and sampling weights were provided in the data sets[245]. Between surveys, the number of states or union territories we included in the analysis the increased from 26 in 1998-99 in to 36, due to the creation of new states from existing ones, for instance, the creation of Jharkhand from Bihar, and Telangana from Andhra Pradesh.

Outcome

In each survey, the participants' height and weight were measured and used to calculate Body Mass Index (BMI). To make the interpretation of our results more straightforward, we categorised the continuous BMI variable using a meaningful qualitative cut-off that facilitate comparison with other studies and adequately capture excess adiposity. Overweight, as well as obese, adults have been reported to be at higher risk of NCDs and all cause-mortality[1718], therefore we categorised individuals as either overweight/obese (BMI over 24.99kg/m^2), or not overweight/obese (BMI less than or equal to 24.99kg/m^2), based on the WHO definition[1]. We additionally used cut-off values recommended for use among Asian populations to verify the trends we initially identified[19], whereby individuals with a BMI greater than 22.99kg/m^2 were classified as overweight/obese, and included the results in the Appendix. Lower BMI cut-off values may be more appropriate among Asian populations, given a potentially higher risk of overweight/obesity related diseases at lower BMI levels compared to populations upon which initial classifications were based[19].

Independent Variables

We considered two measures of SEP: an index of standard of living and educational attainment. It was not possible to include occupation as an independent variable because it was collected on a limited subsample of respondents in the 2015-16 survey.

For peer review only - http://bmjopen.bmj.com/site/about/guidelines.xhtml

We allocated individuals in all the surveys to one of the following four education categories, based on the number of years of schooling: None (0 years); primary (1-5 years); secondary (6-12 years); higher (12+ years). We used Education as a measure of SEP as it may indicate employable skills that expose individuals to more opportunities to earn higher incomes.

The NFHS contains a wealth index, constructed using Principal Components Analysis (PCA) in each survey separately, using information on household asset ownership and household characteristics. As the original wealth index cannot be appropriately compared over time, and as we intended to stratify our analysis by urban and rural areas, we constructed a new index, as an alternative measure of SEP, using PCA from 26 assets and characteristics available in all the surveys[245]. Based on our new wealth scores derived from weightings given to each asset or characteristic, households were classified as either ‘lower’, ‘medium’ or ‘higher’ standard of living (SoL). Asset-based indices are commonly used in cross sectional studies conducted in low and middle-income countries, where income data may be an unreliable indicator of overall SEP, particularly in rural areas[20]. For instance, households may receive income from a variety of sources, which may be difficult to recall, or income may be received in kind[2021] rather than monetarily. Consequently, a household’s stock of assets may provide a more reliable measure of current SEP[20].

We adjusted our final models for the respondent’s age (categorised as 15-29; 30-39, and 40-49 (40-54) for women (men)), as it has been reported in previous studies that overweight/obesity prevalence increases with age[22]. Additionally, older adults may have accumulated more assets over a longer lifespan, potentially, confounding the association between SEP and overweight/obesity. Research has found overweight/obesity to be higher among married individuals, and therefore could confound the reported association between SEP and overweight/obesity.

Statistical Analysis

We initially calculated the prevalence of overweight/obesity in each standard of living index and educational attainment category, by sex and urban/rural residence. We accounted for the complex survey design of the data using sampling weights. Separately for urban and rural areas, we calculated the ratio of the prevalence between the highest and lowest socio-economic status group of our two main SEP variables (eg. higher to lower standard of living, and higher to no education) in each of the surveys. Additionally, we calculated the percentage change in the prevalence of overweight/obesity by each category of standard of living and educational attainment.

Separately for urban and rural areas, and sex, we fitted multilevel logistic regression models with random intercepts for primary sampling units and states. We chose to include PSU- and state-level random intercepts due to the hierarchical nature of the NFHS data, whereby individuals are nested within PSUs, which are nested within states. Standard errors calculated in our models would have been underestimated if we did not account for this clustering. We modelled the log odds ratio of overweight/obesity in each category of the SEP variable

For peer review only

of interest in each of the surveys by fitting a survey specific interaction term. The regression models were adjusted for the covariates mentioned in the independent variables section, in addition to the remaining SEP variable. No evidence of multicollinearity of independent variables with the main exposure of interest was detected when examining changes in the standard error once new variables were added. Finally, we derived and reported the predicted prevalence of overweight/obesity from the model, in addition to their 95% confidence bounds. Adjusted analyses were also carried out using Asian specific BMI cut-offs to observe if the trends identified varied depending on the outcome measure used (Appendix).

Patient and public involvement

Publicly available survey data was used for the analysis and no patients were involved in the study.

Results

The study population generally experienced increasing educational attainment and standard of living over the period of analysis in both urban and rural areas. Whereas the percentage of respondents with no education declined over the study period, particularly among the rural population, the percentage with secondary education in the 2015-16 survey was generally higher than in 1998-99 and 2005-06. Additionally, in both rural and urban areas, the percentage of individuals from lower SoL households declined, whilst the percentage from higher SoL households increased between 1998 and 2016 (Tables 1 and 2).

Table 1. Characteristics of Rural study participants across NFHS surveys with recorded BMI information

	Women						Men			
	NFHS 2 (1998-99)		NFHS 3 (2005-06)		NFHS 4 (2015-16)		NFHS 3 (2005-06)		NFHS 4 (2015-16)	
	Freq	%	Freq	%	Freq	%	Freq	%	Freq	%
Not overweight/obese	49596	0.93	42979	0.9	289482	0.83	32304	0.93	64133	0.86
Overweight/obese	3496	0.07	4912	0.1	61124	0.17	2255	0.07	10550	0.14
Age 15-29	23888	0.45	19279	0.4	126796	0.36	16537	0.48	34589	0.46
Age 30-39	17488	0.33	16892	0.35	122520	0.35	8951	0.26	18965	0.25
Age 40-49(54 males)	11716	0.22	11720	0.24	101290	0.29	9071	0.26	21129	0.28
No Education	31724	0.6	24314	0.51	146302	0.42	6904	0.2	11709	0.16
Primary	9469	0.18	8417	0.18	55652	0.16	6620	0.19	10545	0.14
Secondary	9971	0.19	13872	0.29	131722	0.38	18199	0.53	43737	0.59
Higher	1916	0.04	1285	0.03	16930	0.05	2824	0.08	8692	0.12
Low SoL	28408	0.54	21262	0.44	64998	0.19	14615	0.42	11842	0.17
Middle SoL	18616	0.35	15929	0.33	120050	0.36	12508	0.36	25338	0.35
High SoL	5869	0.11	10645	0.22	149191	0.45	7409	0.21	34202	0.48
Married	49674	0.94	44763	0.93	331883	0.95	22352	0.65	47948	0.64
Not married	3418	0.06	3128	0.07	18723	0.05	12207	0.35	26735	0.36

For peer review only - http://bmjopen.bmj.com/site/about/guidelines.xhtml

Table 2. Characteristics of Urban study participants across NFHS surveys with recorded BMI information

	NFHS 2 (1998-99)		NFHS 3 (2005-06)		NFHS 4 (2015-16)		NFHS 3 (2005-06)		NFHS 4 (2015-16)	
	Freq	%	Freq	%	Freq	%	Freq	%	Freq	%
Not overweight/obese	18473	0.75	25454	0.7	87695	0.64	28669	0.83	25285	0.74
Overweight/obese	6048	0.25	10808	0.3	49443	0.36	5981	0.17	8732	0.26
Age 15-29	8950	0.36	12401	0.34	41893	0.31	17434	0.5	15581	0.46
Age 30-39	9253	0.38	13954	0.38	52032	0.38	8652	0.25	8742	0.26
Age 40-49(54 males)	6318	0.26	9907	0.27	43213	0.32	8564	0.25	9694	0.28
No Education	6493	0.26	9048	0.25	28878	0.21	3016	0.09	2884	0.08
Primary	4025	0.16	4959	0.14	16818	0.12	4143	0.12	3407	0.1
Secondary	8814	0.36	16655	0.46	67583	0.49	19902	0.57	19563	0.58
Higher	5181	0.21	5596	0.15	23859	0.17	7574	0.22	8163	0.24
Low SoL	16444	0.67	17263	0.48	33609	0.25	17329	0.5	8773	0.27
Middle SoL	5682	0.23	10147	0.28	50027	0.38	9613	0.28	11925	0.36
High SoL	2310	0.09	8832	0.24	49540	0.37	7694	0.22	12389	0.37
Married	22931	0.94	33845	0.93	128279	0.94	19656	0.57	20375	0.6
Not married	1590	0.06	2417	0.07	8859	0.06	14994	0.43	13642	0.4

The prevalence of overweight/obesity increased in each successive survey for both of our samples of men and women. In rural India, the prevalence among men almost tripled from 0.059 to 0.148 between 2005 and 2016, and among women, the prevalence increased from 0.059 to 0.182 between 1998 and 2016. In urban India, the prevalence among women increased to 0.385 in 2015-16, from 0.236 in 1998-99, whereas the prevalence among urban men increased from 0.167 to 0.276 between 2005 and 2016 (Figure 1).

Figure 1. Prevalence (weighted) of overweight/obesity in urban and rural India, among men and women

[FIGURE 1 ABOUT HERE]

In all surveys, and for men and women in both urban and rural areas, the prevalence of overweight/obesity was highest among participants with higher education and from a higher SoL, whereas the lowest prevalence of overweight/obesity was found among participants with no education and from a lower SoL.

However, over the study periods for both men and women, the greatest percentage increase in overweight/obesity prevalence was observed among participants from the lowest SoL category and participants with no education. Consequently, the ratio of the prevalence of overweight/obesity in all of the highest, compared to the lowest, SEP groups, reduced over time (Tables 3 and 4).

For peer review only

Table 3. Percentage of respondents classified as overweight/obese, by Education level (1998-2016)

					Women			Men
		1998-99	2005-06	2015-16	% change	2005-06	2015-16	% change
		%	%	%	1998-2016	%	%	2005-2016
Rural	Education**							
	No Education	3.38	5.26	13.91	311.54	3.05	10.79	253.77
	Primary	7.93	10.01	18.45	132.66	4.22	14.06	233.18
	Secondary	10.8	14.19	21.82	102.04	6.57	14.56	121.61
	Higher	15.85	22.79	26.73	68.64	15.32	22.32	45.69
	Ratio*	4.69	4.33	1.92		5.02	2.07	
Urban	Education**							
	No Education	13.53	18.49	32.17	137.77	7.73	18.28	136.48
	Primary	19.45	24.45	37.21	91.31	10.9	23.86	118.90
	Secondary	27.18	33.04	40.15	47.72	15.24	26.33	72.77
	Higher	35.35	41.79	41.56	17.57	28.39	34.87	22.82
	Ratio*	2.61	2.26	1.29		3.67	1.91	

*Ratio of the percentage among individuals with Higher education and no education
** Chi2 test p-value of each strata's association with overweight/obesity p<0.001

Table 4. Percentage of respondents classified as overweight/obese, by Standard of Living (1998-2016)

					Women			Men
		1998-99	2005-06	2015-16	% change	2005-06	2015-16	% change
		%	%	%	1998-2016	%	%	2005-2016
Rural	Standard of Living**							
	Lower SoL	2.35	3.01	6.65	182.98	1.79	4.96	177.09
	Middle SoL	8.22	8.88	12.94	57.42	5.66	9.47	67.31
	Higher SoL	22.93	25.15	27.74	20.98	17.49	22.3	27.50
	Ratio*	9.76	8.36	4.17		9.77	4.50	
Urban	Standard of Living**							
	Lower SoL	16.32	17.36	24.91	52.63	8.92	16.01	79.48
	Middle SoL	39.11	35.01	38.83	-0.72	20.61	26.89	30.47
	Higher SoL	46.93	48.4	46.87	-0.13	30.59	35.77	16.93
	Ratio*	2.88	2.79	1.88		3.43	2.23	

*Ratio of the percentage in the highest and lowest socio-economic group
** Chi2 test p-value of each strata's association with overweight/obesity p<0.001

Figure 2. Predicted prevalence* of overweight/obesity in India, by Educational attainment (1998-2016)

[FIGURE 2 ABOUT HERE]

Figure 3. Predicted prevalence* of overweight/obesity in India, by Standard of Living (1998-2016)

For peer review only

[FIGURE 3 ABOUT HERE]

After adjusting for marital status and age, in urban areas, the predicted prevalence of overweight/obesity among lower SEP women increased over the study period for both men and women, whereas no notable changes were observed among higher SEP women. Among urban men, we observed some increase in the prevalence of overweight/obesity among high SEP respondents, however, the increase among low SEP men was greater. Among both rural men and women, more similar increases were observed among individuals from all SEP groups over the study period (Figures 2 and 3). Equivalent trends were found when using the BMI cut-offs recommended for Asian populations (Figures A1 and A2 in appendix).

Discussion

We found that, although overweight/obesity prevalence increased with SEP, in urban areas no notable change in the prevalence of overweight/obesity was observed among higher SEP women, whereas the prevalence among lower SEP women increased considerably between 1998-2016. The prevalence increase of overweight/obesity was greater among lower SEP urban men compared with higher SEP counterparts between 2005 and 2016. Consequently, some convergence of overweight/obesity across SEP was observed in urban areas among both men and women. In rural areas however, overweight/obesity prevalence increased similarly among individuals in all SEP groups, with fewer signs of convergence across SEP groups yet.

Strengths and limitations

The main strength of our study is our use of the most recent nationally representative data available for India, making our results the most up-to-date estimates of overweight/obesity trends by SEP.

Our study however has some limitations. Firstly, we derive our only measure of overweight/obesity from BMI, rather than complement our results with alternative measures of overweight/obesity, such as waist circumference[23,24] and body fat percentage. Consequently, the prevalence estimates we report may vary depending on the adiposity measure and the exact definitions/cut-offs used. However, given the high correlation between BMI and measures including waist circumference among Indians[25], we would not expect the reported associations between overweight/obesity and SEP, and trends, to change considerably between measures.

Secondly, to ensure the population of sampled women was comparable over time, we limited our analysis to ever-married women, as this was the selection criteria in the NFHS-2 survey. Prevalence of overweight/obesity is generally lower among never-married women[26], for instance in the NFHS-4 survey data, the prevalence of overweight/obesity was 6.6% among never-married women, compared to 25.0% among currently married women. This may have lead us to overestimate overweight/obesity prevalence among women, as the weighted percentage of never-married women were 19.8% and 22.5% in the 2005-06 and 2015-16 samples,

For peer review only - http://bmjopen.bmj.com/site/about/guidelines.xhtml

respectively. However, although individual point estimates may be affected, we do not expect the association between overweight/obesity and SEP we identified to be overestimated.

Our SoL index may also imperfectly capture household wealth. For instance, no indication about the quality of assets used in the measure were included, potentially misclassifying certain households[2027]. However, as three broad SoL groups across a large data set were defined, we do not expect any misclassification to substantially bias our results. Additionally, the association between the true SEP and certain assets included in the SoL index may differ between urban and rural areas. We attempted to account for differences in the value of certain assets by calculating separate indices for urban and rural areas, however, differences in the value of some assets may still exist within broad geographical areas, for instance between states.

Finally, our results may mask variation in subnational prevalence and trends, especially given subnational differences between states in economic growth, demography and culture. For instance, research in India has found that in states with a higher prevalence of overweight, lower and higher SEP group may show a converging risk of overweight/obesity, whereas divergent trends have been identified in states with the highest proportion of underweight individuals[28].

Comparison with other research

The only other India-specific national study we found on this topic did not identify any change in the overweight/obesity-SEP association between 1998-99 and 2005-06 in urban or rural India; with a persisting higher prevalence among high SEP groups[29]. Beyond 2005-06, the authors predicted that future overweight/obesity prevalence would show a similar social patterning as they expected future economic gains to almost solely benefit higher SEP individuals. By contrast, the converging socio-economic patterning of overweight/obesity we have identified in urban areas indicates that economic growth in the past decade may either have been more egalitarian than previously expected, the cost of high calorie food may have become less expensive, or even the pool of susceptible higher SEP individuals may be becoming saturated.

Converging overweight/obesity prevalence between higher and lower SEP groups has been identified sub-nationally in India, when restricted to states defined by a high overall prevalence of overweight[28], mirroring our finding in urban areas. This may suggest that convergence is restricted to areas that have moved beyond the earliest stages of the epidemiological transition.

Though not reported in previous nationally representative studies in India, a converging socioeconomic patterning of overweight/obesity has been noted in some other low and middle-income countries, where the highest increases in overweight prevalence have been found among women working in manual labour[30], among the lowest wealth and income groups[313233] and among rural residents[34].

Potential mechanisms

For peer review only

In rural areas we identified similar increases in prevalence among individuals from all SEP groups. Some studies suggest that in low-income settings, increases in overweight and obesity are restricted to higher SEP individuals, which may be due to changing dietary patterns towards fatty and sugary convenience foods[91011121335], however, the rising prevalence among lower SEP individuals indicates that they may also be increasingly exposed to high calorie foods. Some researchers have also suggested that this mechanism is stronger in low-income or rural settings due to more favourable perceptions of large body sizes across socioeconomic status[13363738].

In urban India, the greater increase in overweight/obesity prevalence among lower SEP individuals mirrors similar findings from places at relatively later stages of economic development, where some researchers have suggested that lower SEP individuals may be priced out of affording relatively expensive low-calorie healthy diets[13394041]. Additionally, lower SEP individuals in urban areas may be more exposed to sedentary lifestyles driven by technological advances replacing manual energy-exerting labour, and improved transport links[4243]. Increased health consciousness, in combination with the ability to afford low calorie diets, may explain why no notable change in overweight/obesity prevalence among the higher SEP urban population was found[134445] in addition to the potential saturation of individuals susceptible to becoming overweight or obese.

Implications

Some studies argue that in India NCD risk factors are almost exclusively an issue for higher SEP individuals[46]. However, our finding that overweight/obesity prevalence has increased among lower SEP individuals in both urban and rural areas implies that to consider overweight/obesity as 'diseases of affluence'[1047] may not be appropriate in India's current context. Efforts to tackle the overall increasing overweight/obesity trend must be inclusive of both the urban and rural poor. This may be especially urgent due to the compounding effect of overweight/obesity and associated NCDs on infectious diseases, which are still highly prevalent among the poor.

Recent initiatives to raise population health include the launch of an integrated National Health Mission[48] which aims to address deficiencies in healthcare delivery across the socioeconomic spectrum in urban and rural areas. Such initiatives may benefit from information about the increasing prevalence among low SEP Indians, as future action aimed at preventing overweight and obesity can be targeted accordingly. Due to the positive association of overweight and obesity with non-communicable diseases such as stroke and diabetes[4950] urgency is required in addressing this modifiable risk factor especially as it could compound existing health complications among poorer Indians, where communicable disease and under-nutrition related diseases already tend to be more prevalent.

Conclusion

Although India is still considered as a lower middle-income country, we have identified some convergence of overweight/obesity prevalence across SEP in urban areas among both men and women, with fewer signs of

For peer review only - http://bmjopen.bmj.com/site/about/guidelines.xhtml

convergence across SEP groups in rural areas. Our findings suggest that an urgent response is needed to slow the increasing trend among poorer Indians, particularly as increasing exposure to overweight and obesity related diseases may compound an already high exposure to infectious diseases.

Conflicts of interest

The authors have no conflicts of interest to declare

Author Contributions

The authors' responsibilities were as follows: Shammi Luhar and Sanjay Kinra designed the study; Shammi Luhar performed the data analysis and takes responsibility for the final content; Shammi Luhar interpreted the results; Shammi Luhar drafted the manuscript; Poppy Mallinson, Lynda Clarke and Sanjay Kinra reviewed and approved the final manuscript.

Data statement

All datasets in this analysis are available at http://www.measuredhs.com.

Funding

This research received no specific grant from any funding agency in the public, commercial or not-for-profit sectors.

Ethical Approval

The analysis of secondary data was approved by the London School of Hygiene and Tropical Medicine's Research ethics committee.

Informed Consent

Data used was anonymised. After receiving detailed information on the survey, participants were asked to give consent to participate in the NFHS surveys by signing a consent declaration.

Acknowledgements

We thank measure DHS for granting us access to the data used in this study.

References

[1] World Health Organization, 2016. Fact Sheet 311: obesity and overweight. Available at: http://www.who.int/mediacentre/factsheets/fs311/en/ [Accessed 13 Jul. 2017].
[2] International Institute for Population Sciences (IIPS) and ORC Macro 2017. National Family Health Survey (NFHS-4), 2014-15: India. Mumbai: IIPS.
[3] DeSA, U.N., 2013. World population prospects: the 2012 revision. Population division of the department of economic and social affairs of the United Nations Secretariat, New York.
[4] International Institute for Population Sciences (IIPS) and Macro International. 2007. National Family Health Survey (NFHS-3), 2005-06: India: Volume I. Mumbai: IIPS.

For peer review only - http://bmjopen.bmj.com/site/about/guidelines.xhtml

[5] International Institute for Population Sciences (IIPS) and ORC Macro 2000. National Family Health Survey (NFHS-2), 1998-99: India. Mumbai: IIPS.

[6] Wang, Y., Chen, H.J., Shaikh, S. and Mathur, P., 2009. Is obesity becoming a public health problem in India? Examine the shift from under-to overnutrition problems over time. Obesity Reviews, 10(4), pp.456-474.

[7] Yadav, S. and Arokiasamy, P., 2014. Understanding epidemiological transition in India. Global health action, 7(1), p.23248.

[8] Reid, C.M. and Thrift, A.G., 2005. Hypertension 2020: confronting tomorrow's problem today. Clinical and experimental pharmacology and physiology, 32(5-6), pp.374-376.

[9] Samal, S., Panigrahi, P. and Dutta, A., 2015. Social epidemiology of excess weight and central adiposity in older Indians: analysis of Study on global AGEing and adult health (SAGE). BMJ open, 5(11), p.e008608.

[10] Monteiro, C.A., Moura, E.C., Conde, W.L. and Popkin, B.M., 2004. Socioeconomic status and obesity in adult populations of developing countries: a review. Bulletin of the World Health Organization, 82(12), pp.940-946.

[11] Gouda, J. and Prusty, R.K., 2014. Overweight and obesity among women by economic stratum in urban India. Journal of health, population, and nutrition, 32(1), p.79.

[12] Kain, J., Vio, F. and Albala, C., 2003. Obesity trends and determinant factors in Latin America. Cadernos de Saude Publica, 19, pp.S77-S86.

[13] Dinsa, G.D., Goryakin, Y., Fumagalli, E. and Suhrcke, M., 2012. Obesity and socioeconomic status in devel- oping countries: a systematic review. Obesity reviews, 13(11), pp.1067-1079.

[14] World Bank, n.d. India. India | Data. URL https://data.worldbank.org/country/india (accessed 4.27.18).

[15] International Monetary Fund., 2018. GDP, current prices. IMF DataMapper. URL http://www.imf.org/external/datamapper/NGDPD@WEO/OEMDC/ADVEC/WEOWORLD/IND (accessed 4.27.18).

[16] Traill, W.B., 2006. Trends towards overweight in lower-and middle-income countries: some causes and economic policy options. The double burden of malnutrition, p.305.

[17] Di Angelantonio, E., Bhupathiraju, S.N., Wormser, D., Gao, P., Kaptoge, S., de Gonzalez, A.B., Cairns, B.J., Huxley, R., Jackson, C.L., Joshy, G. and Lewington, S., 2016. Body-mass index and all-cause mortality: individual-participant-data meta-analysis of 239 prospective studies in four continents. The Lancet, 388(10046), pp.776-786.

[18] Chen, Y., Copeland, W.K., Vedanthan, R., Grant, E., Lee, J.E., Gu, D., Gupta, P.C., Ramadas, K., Inoue, M., Tsugane, S. and Tamakoshi, A., 2013. Association between body mass index and cardiovascular disease mortality in east Asians and south Asians: pooled analysis of prospective data from the Asia Cohort Consortium

[19] WHO, E.C., 2004. Appropriate body-mass index for Asian populations and its implications for policy and intervention strategies. Lancet (London, England), 363(9403), p.157.

[20] Vyas, S. and Kumaranayake, L., 2006. Constructing socio-economic status indices: how to use principal components analysis. Health policy and planning, 21(6), pp.459-468.

[21] McKenzie, D.J., 2003. Measure inequality with asset indicators. Bureau for Research and Economic Analysis of Development, Centre for International Development, BREAD.

[22] Subramanian, S.V., Kawachi, I. and Smith, G.D., 2007. Income inequality and the double burden of under-and overnutrition in India. Journal of Epidemiology & Community Health, 61(9), pp.802-809.

[23] Misra, A., Vikram, N.K., Gupta, R., Pandey, R.M., Wasir, J.S. and Gupta, V.P., 2006. Waist circumference cutoff points and action levels for Asian Indians for identification of abdominal obesity. *International journal of obesity*, *30*(1), p.106.

[24] Subramanian, S.V. and Smith, G.D., 2006. Patterns, distribution, and determinants of under-and overnutrition: a population-based study of women in India. The American Journal of clinical nutrition, 84(3), pp.633-640.

[25] Kuper, H., Taylor, A., Krishna, K.V.R., Ben-Shlomo, Y., Gupta, R., Kulkarni, B., Prabhakaran, D., Smith, G.D., Wells, J., Ebrahim, S. and Kinra, S., 2014. Is vulnerability to cardiometabolic disease in Indians mediated by abdominal adiposity or higher body adiposity. BMC public health, 14(1), p.1239.

[26] Lipowicz, A., Gronkiewicz, S. and Malina, R.M., 2002. Body mass index, overweight and obesity in married and never married men and women in Poland. American Journal of Human Biology, 14(4), pp.468-475.

[27] Falkingham, J. and Namazie, C., 2002. Measuring health and poverty: a review of approaches to identifying the poor. London: DFID Health Systems Resource Centre, p.7.

[28] Sengupta, A., Angeli, F., Syamala, T.S., Dagnelie, P.C. and van Schayck, C.P., 2015. Overweight and obesity prevalence among Indian women by place of residence and socio-economic status: Contrasting patterns from 'underweight states' and 'overweight states' of India. Social Science & Medicine, 138, pp.161-169.

[29] Subramanian, S.V., Perkins, J.M. and Khan, K.T., 2009. Do burdens of underweight and overweight coexist among lower socioeconomic groups in India?–. The American journal of clinical nutrition, 90(2), pp.369-376.

[30] Lopez-Arana, S., Avendano, M., van Lenthe, F.J. and Burdorf, A., 2014. Trends in overweight among women differ by occupational class: results from 33 low-and middle-income countries in the period 1992–2009. International Journal of Obesity, 38(1), p.97.

[31] Jones-Smith, J.C., Gordon-Larsen, P., Siddiqi, A. and Popkin, B.M., 2012. Is the burden of overweight shifting to the poor across the globe? Time trends among women in 39 low-and middle-income countries (1991–2008). International journal of obesity, 36(8), p.1114.

[32] Monteiro, C.A., Conde, W.L. and Popkin, B.M., 2007. Income-specific trends in obesity in Brazil: 1975–2003. American journal of public health, 97(10), pp.1808-1812.

For peer review only

For peer review only - http://bmjopen.bmj.com/site/about/guidelines.xhtml

[33] Nguyen, M.D., Beresford, S.A. and Drewnowski, A., 2007. Trends in overweight by socio-economic status in Vietnam: 1992 to 2002. Public health nutrition, 10(2), pp.115-121.
[34] Wang, H., Du, S., Zhai, F. and Popkin, B.M., 2007. Trends in the distribution of body mass index among Chinese adults, aged 20–45 years (1989–2000). International Journal of Obesity, 31(2), p.272.
[35] Khandelwal, S. and Reddy, K.S., 2013. Eliciting a policy response for the rising epidemic of overweight-obesity in India. obesity reviews, 14(S2), pp.114-125.
[36] Rguibi, M. and Belahsen, R., 2006. Body size preferences and sociocultural influences on attitudes towards obesity among Moroccan Sahraoui women. Body Image, 3(4), pp.395-400.
[37] Fernald, L.C., 2009. Perception of body weight: a critical factor in understanding obesity in middle-income countries. Journal of Womens Health, 18(8), pp.1121-1122.
[38] Gulati S, Misra A, Colles S et al. 2013. Dietary intakes and familial correlates of overweight/obesity: a four cities study in India. Annals of Nutrition and Metabolism 62: 279–90.
[39] Temple, N.J., Steyn, N.P., Fourie, J. and De Villiers, A., 2011. Price and availability of healthy food: A study in rural South Africa. Nutrition, 27(1), pp.55-58.
[40] Ramachandran, P., 2006. The double burden of malnutrition in India. Rome: FAO Food and Nutrition Papers - Food and Agriculture Organization of the United Nations, pp.99-160.
[41] Uustialo, U., Pietinen, P. & Puska, P., 2002. Dietary transition in developing countries: challenges for chronic disease prevention. In WHO. Globalization, diets and non-communicable diseases. Geneva, WHO. 25.
[42] Singh, R.B., Pella, D., Mechirova, V., Kartikey, K., Demeester, F., Tomar, R.S., Beegom, R., Mehta, A.S., Gupta, S.B., De, A.K. and Neki, N.S., 2007. Prevalence of obesity, physical inactivity and undernutrition, a triple burden of diseases during transition in a developing economy. The Five City Study Group. Acta cardiologica, 62(2), p.119.
[43] Unnikrishnan, A.G., Kalra, S. and Garg, M.K., 2012. Preventing obesity in India: Weighing the options. Indian journal of endocrinology and metabolism, 16(1), p.4.
[44] Lallukka T., Laaksonen M., Rahkonen O., Roos E., Lahelma E. 2009. Multiple socio-economic circumstances and healthy food habits. Cent. Eur. J. Public Health 17, 55–63
[45] Kearney, J., 2010. Food consumption trends and drivers. Philosophical transactions of the royal society B: biological sciences, 365(1554), pp.2793-2807.
[46] Subramanian, S.V., Corsi, D.J., Subramanyam, M.A. and Davey Smith, G., 2013. Jumping the gun: the problematic discourse on socioeconomic status and cardiovascular health in India. International journal of epidemiology, 42(5), pp.1410-1426.
[47] Ezzati, M., Vander Hoorn, S., Lawes, C.M., Leach, R., James, W.P.T., Lopez, A.D., Rodgers, A. and Murray, C.J., 2005. Rethinking the “diseases of affluence” paradigm: global patterns of nutritional risks in relation to economic development. PLoS medicine, 2(5), p.e133.
[48] D'Silva, J., 2013. Can India pull off its ambitious National Health Mission?. BMJ: British Medical Journal (Online), 346.
[49] Kivimäki, M., Kuosma, E., Ferrie, J.E., Luukkonen, R., Nyberg, S.T., Alfredsson, L., Batty, G.D., Brunner, E.J., Fransson, E., Goldberg, M. and Knutsson, A., 2017. Overweight, obesity, and risk of cardiometabolic multimorbidity: pooled analysis of individual-level data for 120 813 adults from 16 cohort studies from the USA and Europe. The Lancet Public Health, 2(6), pp.e277-e285.
[50] Babu, G.R., Murthy, G.V.S., Ana, Y., Patel, P., Deepa, R., Neelon, S.E.B., Kinra, S. and Reddy, K.S., 2018. Association of obesity with hypertension and type 2 diabetes mellitus in India: A meta-analysis of observational studies. World Journal of Diabetes, 9(1), p.40.

For peer review only

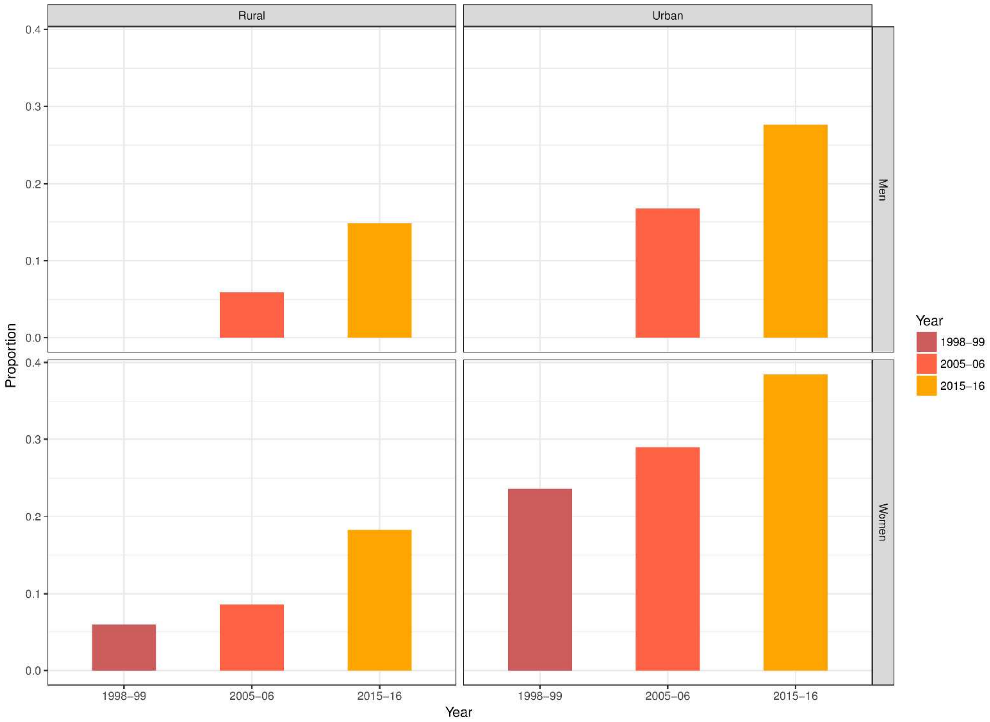

For peer review only

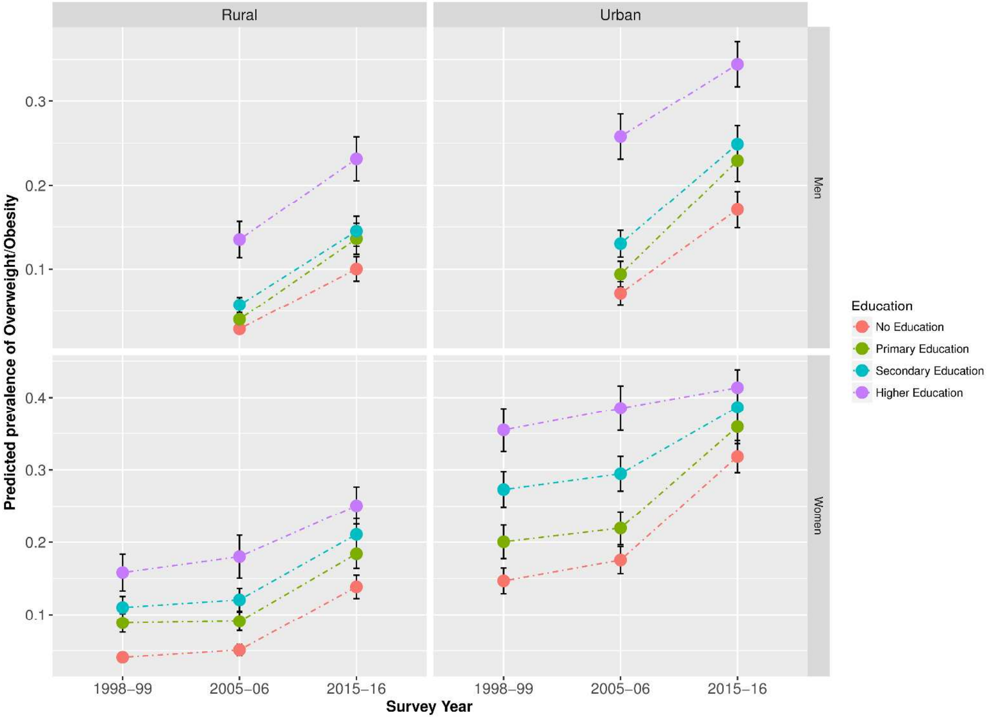

*Predicted prevalences and confidence intervals are based on multivariate regressions, and the models adjust for the respondent's age, current marital status and the socio-economic variable not considered as the main exposure.

For peer review only - http://bmjopen.bmj.com/site/about/guidelines.xhtml

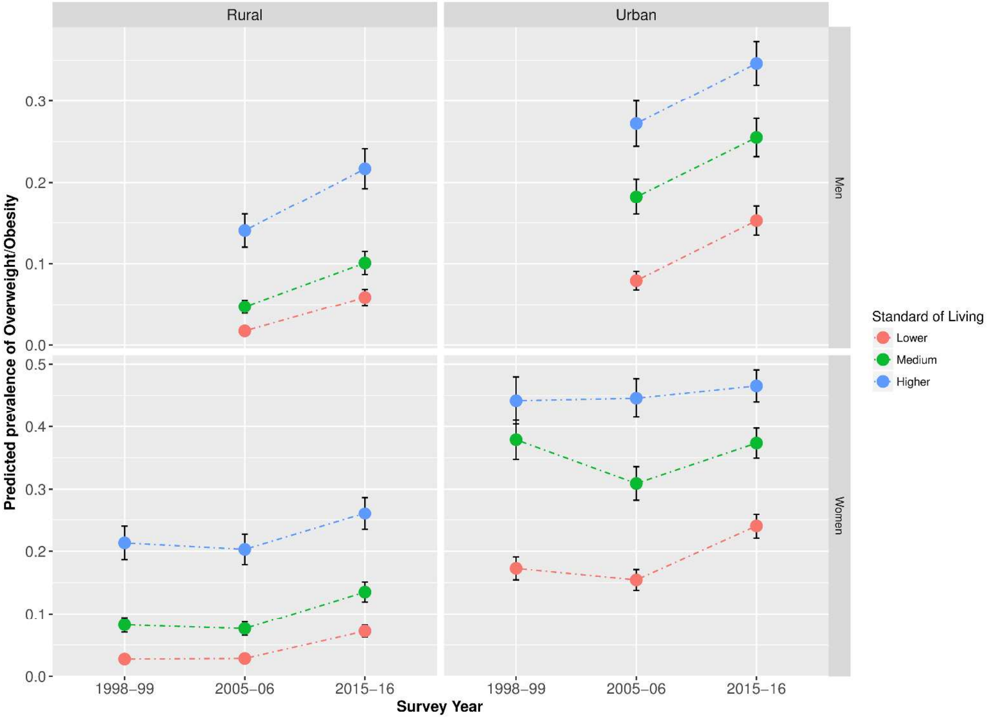

*Predicted prevalences and confidence intervals are based on multivariate regressions, and the models adjust for the respondent's age, current marital status and the socio-economic variable not considered as the main exposure.

For peer review only

Appendix

Figure A1. Predicted prevalence* of overweight/obesity in India by Education (using South Asian BMI cut-offs) (1998-2016)

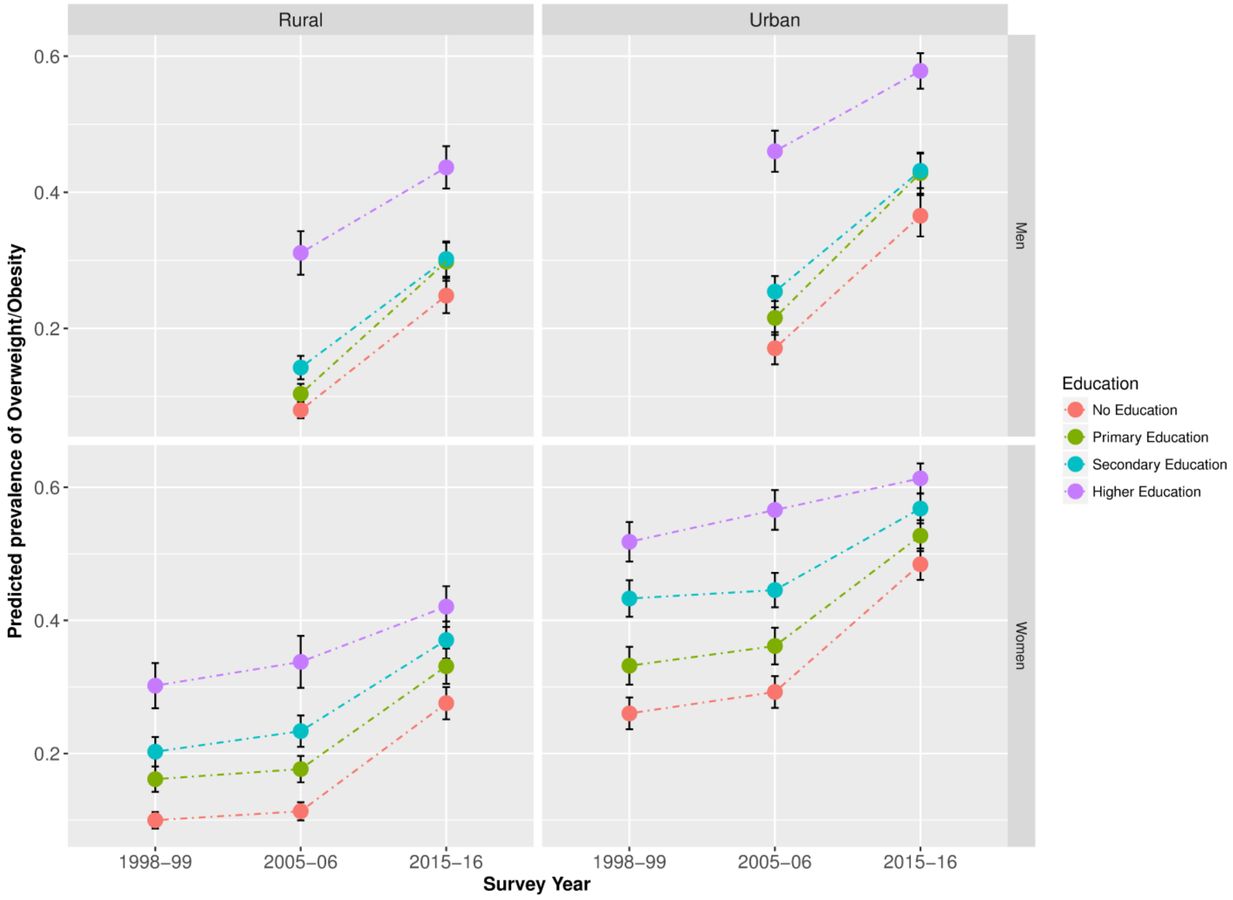

Figure A2. Predicted prevalence* of overweight/obesity in India by Standard of Living (using South Asian BMI cut-offs) (1998-2016)

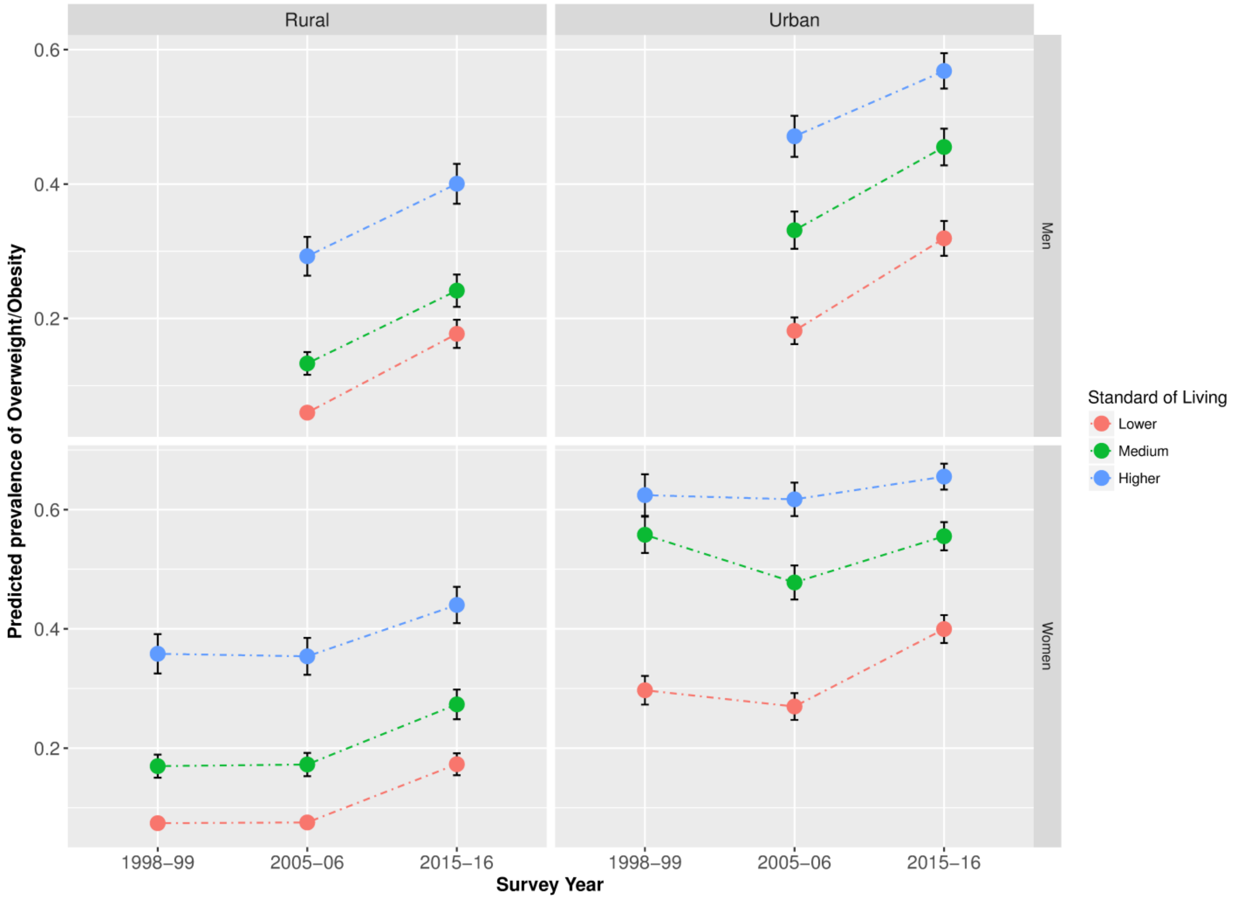

Reporting checklist for cross sectional study.

Based on the STROBE cross sectional guidelines.

Instructions to authors

Complete this checklist by entering the page numbers from your manuscript where readers will find each of the items listed below.

Your article may not currently address all the items on the checklist. Please modify your text to include the missing information. If you are certain that an item does not apply, please write "n/a" and provide a short explanation.

Upload your completed checklist as an extra file when you submit to a journal.

In your methods section, say that you used the STROBE cross sectional reporting guidelines, and cite them as:

von Elm E, Altman DG, Egger M, Pocock SJ, Gotzsche PC, Vandenbroucke JP. The Strengthening the Reporting of Observational Studies in Epidemiology (STROBE) Statement: guidelines for reporting observational studies.

		Reporting Item	Page Number
Title	#1a	Indicate the study's design with a commonly used term in the title or the abstract	1
Abstract	#1b	Provide in the abstract an informative and balanced summary of what was done and what was found	2
Background / rationale	#2	Explain the scientific background and rationale for the investigation being reported	3
Objectives	#3	State specific objectives, including any prespecified hypotheses	3
Study design	#4	Present key elements of study design early in the paper	4
Setting	#5	Describe the setting, locations, and relevant dates, including periods of recruitment, exposure, follow-up, and data collection	4
Eligibility criteria	#6a	Give the eligibility criteria, and the sources and methods of selection of participants.	4

For peer review only

	#7	Clearly define all outcomes, exposures, predictors, potential confounders, and effect modifiers. Give diagnostic criteria, if applicable	4
Data sources / measurement	#8	For each variable of interest give sources of data and details of methods of assessment (measurement). Describe comparability of assessment methods if there is more than one group. Give information separately for for exposed and unexposed groups if applicable.	4
Bias	#9	Describe any efforts to address potential sources of bias	4
Study size	#10	Explain how the study size was arrived at	4
Quantitative variables	#11	Explain how quantitative variables were handled in the analyses. If applicable, describe which groupings were chosen, and why	4
Statistical methods	#12a	Describe all statistical methods, including those used to control for confounding	5
	#12b	Describe any methods used to examine subgroups and interactions	5
	#12c	Explain how missing data were addressed	4
	#12d	If applicable, describe analytical methods taking account of sampling strategy	5
	#12e	Describe any sensitivity analyses	5
Participants	#13a	Report numbers of individuals at each stage of study—eg numbers potentially eligible, examined for eligibility, confirmed eligible, included in the study, completing follow-up, and analysed. Give information separately for for exposed and unexposed groups if applicable.	4
	#13b	Give reasons for non-participation at each stage	4
	#13c	Consider use of a flow diagram	4
Descriptive data	#14a	Give characteristics of study participants (eg demographic, clinical, social) and information on exposures and potential confounders. Give information separately for exposed and unexposed groups if applicable.	6

For peer review only

	#14b	Indicate number of participants with missing data for each variable of interest	4
Outcome data	#15	Report numbers of outcome events or summary measures. Give information separately for exposed and unexposed groups if applicable.	6
Main results	#16a	Give unadjusted estimates and, if applicable, confounder-adjusted estimates and their precision (eg, 95% confidence interval). Make clear which confounders were adjusted for and why they were included	7,8
	#16b	Report category boundaries when continuous variables were categorized	8
	#16c	If relevant, consider translating estimates of relative risk into absolute risk for a meaningful time period	8
Other analyses	#17	Report other analyses done—e.g., analyses of subgroups and interactions, and sensitivity analyses	7,8
Key results	#18	Summarise key results with reference to study objectives	8
Limitations	#19	Discuss limitations of the study, taking into account sources of potential bias or imprecision. Discuss both direction and magnitude of any potential bias.	8,9
Interpretation	#20	Give a cautious overall interpretation considering objectives, limitations, multiplicity of analyses, results from similar studies, and other relevant evidence.	9,10
Generalisability	#21	Discuss the generalisability (external validity) of the study results	9
Funding	#22	Give the source of funding and the role of the funders for the present study and, if applicable, for the original study on which the present article is based	11

The STROBE checklist is distributed under the terms of the Creative Commons Attribution License CC-BY. This checklist was completed on 01. May 2018 using http://www.goodreports.org/, a tool made by the EQUATOR Network in collaboration with Penelope.ai

For peer review only
